# Characterization of influenza A(H1N1)pdm09 viruses isolated from Nepalese and Indian outbreak patients in early 2015

**DOI:** 10.1111/irv.12469

**Published:** 2017-08-09

**Authors:** Kazuya Nakamura, Masayuki Shirakura, Seiichiro Fujisaki, Noriko Kishida, David F. Burke, Derek J. Smith, Tomoko Kuwahara, Emi Takashita, Ikuyo Takayama, Mina Nakauchi, Mandeep Chadha, Varsha Potdar, Arvind Bhushan, Bishnu Prasad Upadhyay, Geeta Shakya, Takato Odagiri, Tsutomu Kageyama, Shinji Watanabe

**Affiliations:** ^1^ Influenza Virus Research Center National Institute of Infectious Diseases Tokyo Japan; ^2^ Center for Pathogen Evolution University of Cambridge Cambridge UK; ^3^ Influenza group National Institute of Virology Indian Council of Medical Research Pune India; ^4^ Department of Health Services National Public Health Laboratory Kathmandu Nepal

**Keywords:** India, influenza A(H1N1)pdm09 virus, Nepal, outbreak

## Abstract

We characterized influenza A(H1N1)pdm09 isolates from large‐scale outbreaks that occurred in Nepal and India in early 2015. Although no specific viral features, which may have caused the outbreaks, were identified, an S84N substitution in hemagglutinin was frequently observed. Chronological phylogenetic analysis revealed that these Nepalese and Indian viruses possessing the S84N substitution constitute potential ancestors of the novel genetic subclade 6B.1 virus that spread globally in the following (2015/16) influenza season. Thus, active surveillance of circulating influenza viruses in the Southern Asia region, including Nepal and India, would be beneficial for detecting novel variant viruses prior to their worldwide spread.

Despite the worldwide predominance of the A(H3N2) virus in the 2014/15 season, India reported a huge outbreak of influenza A(H1N1)pdm09 virus (H1N1pdm) infection with severe clinical outcomes at the beginning of 2015; over 30 000 cases were reported with over 2000 deaths, compared with 5044, 5250 and <1000 reported cases in 2012, 2103, and 2014, respectively.^1^, ^2^, ^3^ Subsequently, Nepal also experienced a regional outbreak with an increased number of patients infected with H1N1pdm (personal communication).^4^ Although a number of Indian case reports have proposed several possible causes for the outbreak and characterized viruses isolated from the patients,^2^, ^5^, ^6^ the viral properties of isolates from outbreak patients remain largely unknown. Thus, in this study, the antigenicity, genetic properties, and antiviral susceptibility of 43 Nepalese isolates from 50 clinical specimens of Nepalese outbreak patients (patient information is summarized in Table S1) and 10 Indian isolates were characterized to identify particular features related to outbreaks with severe and fatal cases.

The antigenicity of these Nepalese and Indian isolates was characterized using the hemagglutination inhibition (HI) test. The hemagglutination activity of all isolates tested was inhibited by ferret antisera raised against several reference viruses including the 2014/15 season vaccine strain, egg‐propagated A/California/07/2009, the high‐growth reassortant strain, A/California/07/2009 (X‐179A), representative field isolates, cell‐propagated A/Narita/1/2009, and A/Wakayama/153/2013 or A/Yokohama/50/2015. The HI titers of these antisera against the Nepalese and Indian isolates were equivalent to or within a fourfold reduction compared to the homologous titer of each reference virus (data not shown), indicating that all Nepalese and Indian isolates tested were antigenically similar to the contemporary circulating H1N1pdm.

The susceptibility of the isolates to four neuraminidase (NA) inhibitors, oseltamivir, peramivir, zanamivir, and laninamivir, was determined using a fluorescent NA inhibition assay with the NA‐Fluor influenza neuraminidase assay kit (Applied Biosystems, Foster City, CA, USA), as previously described.^7^ The results showed that the Nepalese and Indian isolates were susceptible to all four inhibitors (data not shown), suggesting that no antiviral‐resistant viruses had spread in these outbreaks.

Taken together, our viral antigenicity and antiviral susceptibility analyses did not identify any unusual features in the Nepalese and Indian outbreak isolates.

Genetic sequencing and phylogenetic analysis were conducted for all Nepalese and Indian isolates. As shown in Figure 1, the isolates contain a hemagglutinin (HA) gene belonging to genetic clade 6B represented by globally circulating H1N1pdm.^8^ Interestingly, 70% of the Nepalese viruses and all Indian viruses, except for one (A/India/P1510025/2015), formed a subclade branch with a high bootstrap value (92/100) due to an S84N amino acid substitution in their HA (designated as subclade 1 in Figure 1). Of the remaining viruses analyzed (15 Nepalese viruses and one Indian virus), nine Nepalese viruses and one Indian virus formed a small cluster (94/100 bootstrap value). Within this small cluster, five Nepalese viruses formed a subcluster with an E506D amino acid substitution (88/100 bootstrap value; designated as subclade 2 in Figure 1). The other six Nepalese viruses belonged to a different cluster possessing an E491G substitution; within this cluster, five viruses formed a subcluster exhibiting an N129D substitution (87/100 of bootstrap value; designated as subclade 3 in Figure 1). Analysis of the NA gene showed that the Nepalese and Indian viruses possessing an S84N substitution in HA formed a subclade exhibiting V13I and I314M substitutions in NA (designated as subclade 1 in Fig. S1). The remaining viruses formed NA subclades related to the HA subclades (designated as subclade 2 and subclade 3, respectively, in Fig. S1). Although the biological significance of these amino acid substitutions in HA and NA has yet to be precisely elucidated, they seem to be correlated with a certain extent in terms of phylogenetic tree (sub)clade structure, suggesting the coevolution of HA and NA with viral fitness.^9^ The Indian isolate, A/India/P152122/2015, possessed a D222G/N amino acid substitution in its HA. This polymorphism in HA has been occasionally associated with viral pathogenesis.^10^ However, because this is the only isolate analyzed in this study that had this substitution, it is unlikely that this substitution was solely responsible for the outbreaks. The nucleotide sequences of the internal genes (PA, PB1, PB2, NP, M, and NS) of all Indian and seven randomly selected Nepalese isolates were determined using next‐generation sequencing with the Illumina MiSeq system (Illumina K. K., Tokyo, Japan). Genetic and phylogenetic analysis of these genes revealed that most of the Nepalese and Indian isolates were clustered into a genetic subclade separated from other subclades formed by globally circulating H1N1pdm (Fig. S2). The amino acid substitutions identified in the internal genes analyzed are summarized in Table S2. Although several amino acid substitutions accumulated in these isolates compared to the globally circulating H1N1pdm, no known amino acid substitutions related to viral pathogenicity were identified. In addition, there were no characteristic differences in the genetic features of the internal genes between the patients with severe and mild outcomes (Table S2). Sequence data determined in this study were deposited with the GISAID database under accession numbers EPI610408 to EPI610535, EPI630406 to EPI630425, and EPI635481 to EPI635540.

**Figure 1:**
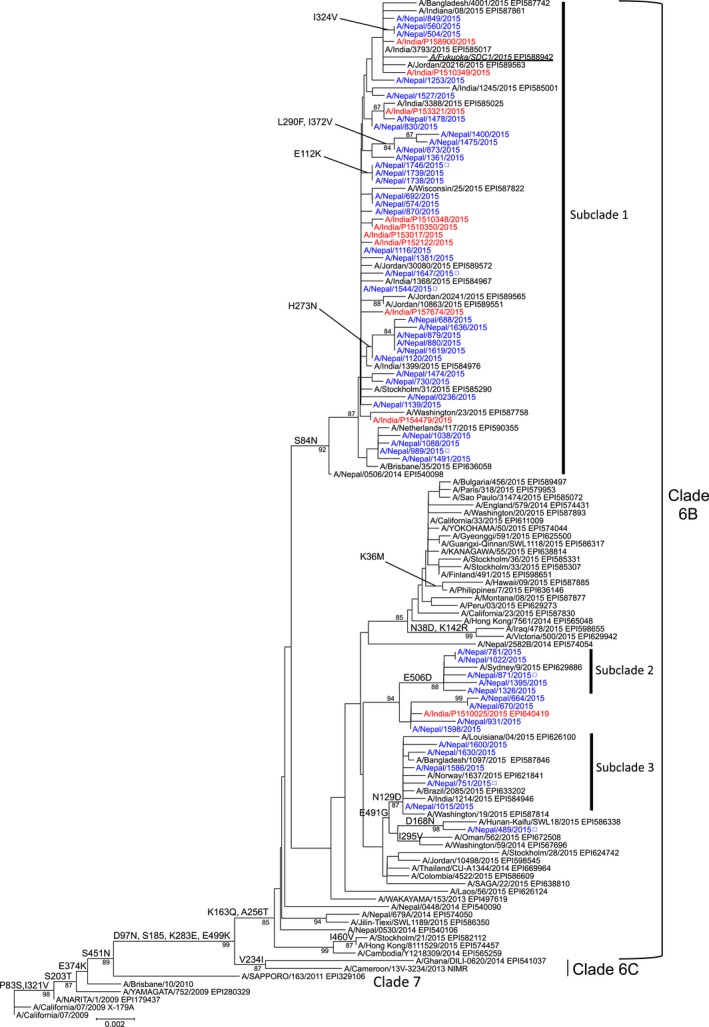
Phylogenetic analysis of the hemagglutinin gene of H1N1pdm strains. Nucleotide sequence data of the former representative H1N1pdm strains and recent 2014/15 season H1N1pdm isolates from around the world were randomly selected from the GISAID database and used as the references. The phylogenetic trees were constructed using the MEGA6 software with the neighbor‐joining method. Nepalese and Indian strains analyzed in this study are colored in blue and red, respectively. Open squares indicate that only genetic sequence data were available in this study because of virus isolation failure. Previously reported^8^ strain A/Fukuoka/SDC1/2015 is underlined. Amino acid substitutions relative to the A/California/07/2009 virus and high bootstrap values (>80) are shown to the left of the nodes. The scale bar indicates the nucleotide substitutions per site. The GISAID EpiFlu database accession numbers for the sequence data of each reference strain are shown

In the following influenza season (2015/16), H1N1pdm infections were more prevalent than A/H3N2 and type B virus infections in the Northern Hemisphere temperate regions, as reported by GISRS.^11^ Most of these H1N1pdm isolates reacted well with the ferret antisera described above (data not shown), indicating that no apparent antigenic change had occurred in the circulating H1N1pdm strains. To determine the genetic correlation between these predominate 2015/16 season H1N1pdm strains and the Nepalese and Indian isolates, further phylogenic analysis with a large number of H1N1pdm strains was conducted (Figure 2). The vast majority of the 2015/16 season H1N1pdm strains (designated by a blue vertical line in Figure 2) formed part of a large cluster possessing an S84N substitution in HA (isolates colored in light blue in Figure 2). These isolates were descended from the Nepalese and Indian isolates described above (designated by a green vertical line in Figure 2). Most of these 2015/16 season isolates contained S162N (on the Sa antigenic site of HA resulting in a potential glycosylation site) and I216T amino acid substitutions in addition to the S84N substitution and formed a subclade subsequently designated as 6B.1.^12^ Intriguingly, this set of S84N variants was preceded by the emergence of isolates possessing an S84N substitution in Nepal in March and April 2014 (designated by a red rectangle in Figure 2 and enlarged in Fig. S3). Although isolates with an S84N substitution were occasionally observed earlier than 2014 in other parts of the world (colored in light blue from 2009 to 2013 in Figure 2), they disappeared shortly thereafter for unknown reasons. The 2014/15 season Nepalese and Indian isolates possessing an S84N substitution originated from isolates with S84N that emerged in Nepal in early 2014 and contributed to further worldwide spread (2015/16 season), suggesting the importance of these areas as a source for the evolution and global spread of novel variant viruses.

**Figure 2:**
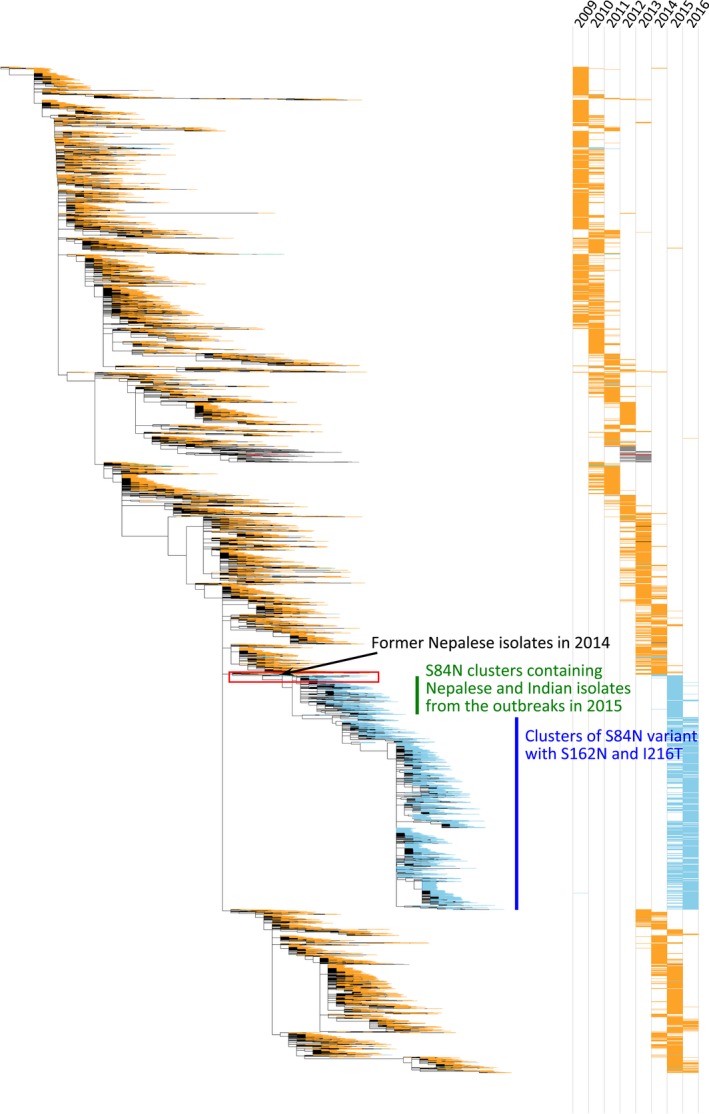
Phylogenetic relationships of the hemagglutinin (HA) genes of Nepalese and Indian isolates and H1N1pdm isolates since 2009. Nucleotide sequence data of H1N1pdm isolates from around the world were obtained from the GISAID database and aligned using MAFFT (available from http://www.ebi.ac.uk/mafft/). MODELTEST was run on the aligned sequences, and GTR+I+gamma4 was determined as the most appropriate evolutionary model for phylogenetic tree construction. The tree was constructed using RAxML v8^13^ according to the GTR+I+gamma4 model. Global optimization of branch topology was performed on the tree with the best likelihood score from RAxML using Garli v0.951 with a run of 150 000 generations. Isolates possessing an S84N substitution in HA are colored in light blue. Isolates possessing S84, S84G, and a mixed population of amino acids are colored in orange, gray, and black, respectively

Although no viral features related to expanded infection with severe and fatal outcomes, including antigenicity, genetic properties, or susceptibility to antivirals, were identified in this study, it is important to note that many Nepalese and Indian H1N1pdm isolates possessing a novel genetic feature (S84N substitution in HA) preceded strains with additional substitutions observed in the subsequent annual influenza season (2015/16 season) in the Northern Hemisphere temperate regions. These novel H1N1pdm variants have also caused widespread outbreaks with severe or fatal outcomes.^11^ The phylogenetic analyses in our current study suggest that South Asian countries, such as Nepal and India, might serve as an important source of variant viruses, and improve our understanding of the evolutionary dynamics of circulating influenza viruses. Thus, the future establishment of systematic measures for monitoring circulating influenza viruses in these countries would be of great importance.

## CONFLICT OF INTEREST

The authors declare that no conflict of interest exists.
