# Supplementary material for: Characterization of influenza A(H1N1)pdm09 viruses isolated from Nepalese and Indian outbreak patients in early 2015
Source: Influenza Other Respir Viruses. 2017 Aug 9;11(5):399–403. doi: 10.1111/irv.12469 (PMC5596518; doi:10.1111/irv.12469)
Supplement: Supplementary file 1 [file IRV-11-399-s001.pptx]

## Slide 1
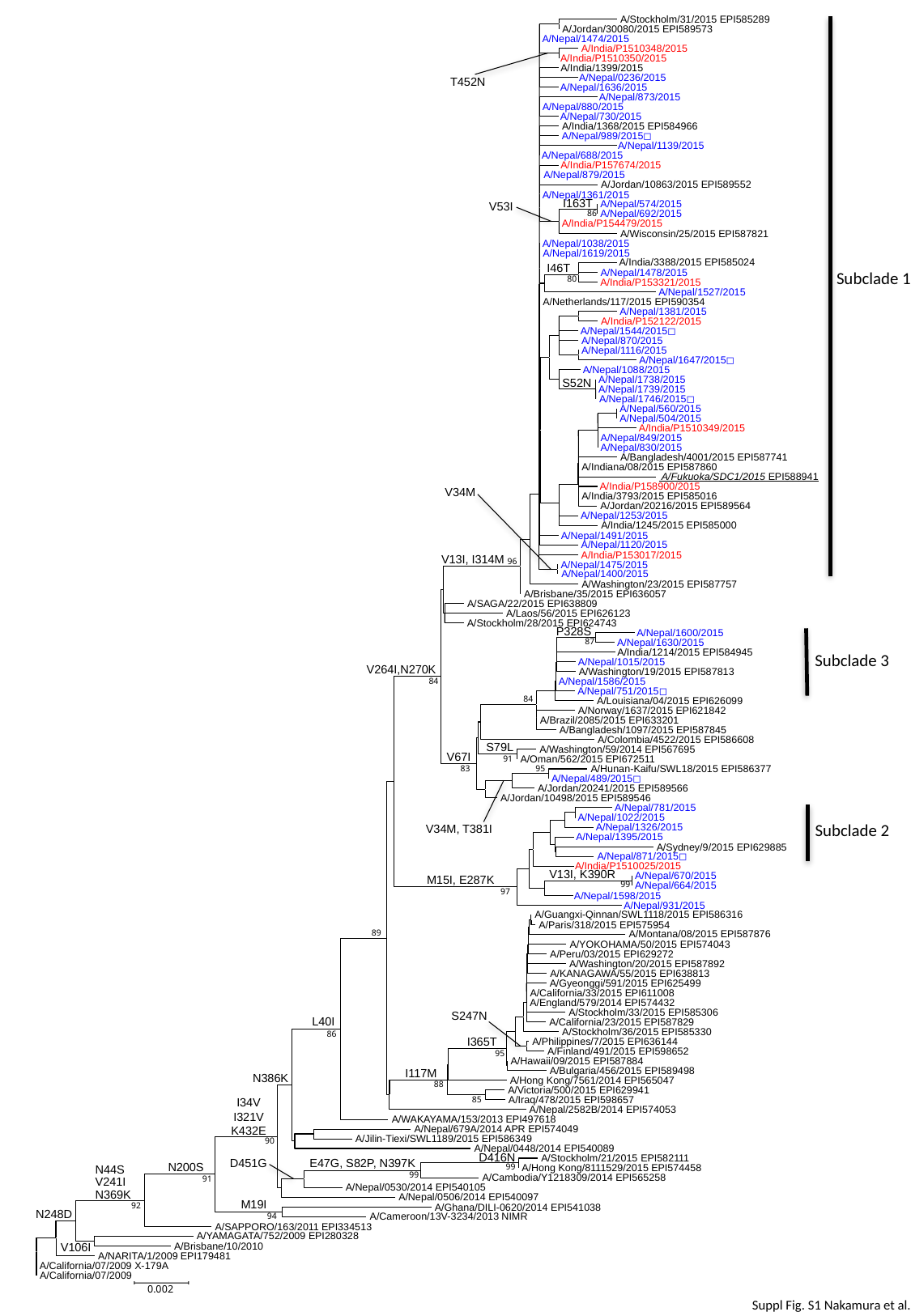

A/Stockholm/31/2015 EPI585289
 A/Jordan/30080/2015 EPI589573
A/Nepal/1474/2015
A/India/P1510348/2015
A/India/P1510350/2015
 A/India/1399/2015
T452N
A/Nepal/0236/2015
A/Nepal/1636/2015
A/Nepal/873/2015
A/Nepal/880/2015
A/Nepal/730/2015
 A/India/1368/2015 EPI584966
A/Nepal/989/2015◻︎
A/Nepal/1139/2015
A/Nepal/688/2015
 A/India/P157674/2015
A/Nepal/879/2015
 A/Jordan/10863/2015 EPI589552
A/Nepal/1361/2015
I163T
V53I
A/Nepal/574/2015
A/Nepal/692/2015
86
A/India/P154479/2015
 A/Wisconsin/25/2015 EPI587821
A/Nepal/1038/2015
A/Nepal/1619/2015
I46T
 A/India/3388/2015 EPI585024
Subclade 1
A/Nepal/1478/2015
80
A/India/P153321/2015
A/Nepal/1527/2015
 A/Netherlands/117/2015 EPI590354
A/Nepal/1381/2015
A/India/P152122/2015
A/Nepal/1544/2015◻︎
A/Nepal/870/2015
A/Nepal/1116/2015
A/Nepal/1647/2015◻︎
A/Nepal/1088/2015
S52N
A/Nepal/1738/2015
A/Nepal/1739/2015
A/Nepal/1746/2015◻︎
A/Nepal/560/2015
A/Nepal/504/2015
A/India/P1510349/2015
A/Nepal/849/2015
A/Nepal/830/2015
 A/Bangladesh/4001/2015 EPI587741
 A/Indiana/08/2015 EPI587860
 A/Fukuoka/SDC1/2015 EPI588941
V34M
A/India/P158900/2015
 A/India/3793/2015 EPI585016
 A/Jordan/20216/2015 EPI589564
A/Nepal/1253/2015
 A/India/1245/2015 EPI585000
A/Nepal/1491/2015
A/Nepal/1120/2015
V13I, I314M
A/India/P153017/2015
96
A/Nepal/1475/2015
A/Nepal/1400/2015
 A/Washington/23/2015 EPI587757
 A/Brisbane/35/2015 EPI636057
 A/SAGA/22/2015 EPI638809
 A/Laos/56/2015 EPI626123
 A/Stockholm/28/2015 EPI624743
P328S
A/Nepal/1600/2015
A/Nepal/1630/2015
87
Subclade 3
 A/India/1214/2015 EPI584945
A/Nepal/1015/2015
V264I,N270K
 A/Washington/19/2015 EPI587813
A/Nepal/1586/2015
84
A/Nepal/751/2015◻︎
84
 A/Louisiana/04/2015 EPI626099
 A/Norway/1637/2015 EPI621842
 A/Brazil/2085/2015 EPI633201
 A/Bangladesh/1097/2015 EPI587845
 A/Colombia/4522/2015 EPI586608
S79L
 A/Washington/59/2014 EPI567695
V67I
 A/Oman/562/2015 EPI672511
91
 A/Hunan-Kaifu/SWL18/2015 EPI586377
83
95
A/Nepal/489/2015◻︎
 A/Jordan/20241/2015 EPI589566
 A/Jordan/10498/2015 EPI589546
A/Nepal/781/2015
A/Nepal/1022/2015
Subclade 2
V34M, T381I
A/Nepal/1326/2015
A/Nepal/1395/2015
 A/Sydney/9/2015 EPI629885
A/Nepal/871/2015◻︎
A/India/P1510025/2015
V13I, K390R
M15I, E287K
A/Nepal/670/2015
A/Nepal/664/2015
99
97
A/Nepal/1598/2015
A/Nepal/931/2015
 A/Guangxi-Qinnan/SWL1118/2015 EPI586316
 A/Paris/318/2015 EPI575954
89
 A/Montana/08/2015 EPI587876
 A/YOKOHAMA/50/2015 EPI574043
 A/Peru/03/2015 EPI629272
 A/Washington/20/2015 EPI587892
 A/KANAGAWA/55/2015 EPI638813
 A/Gyeonggi/591/2015 EPI625499
 A/California/33/2015 EPI611008
 A/England/579/2014 EPI574432
S247N
 A/Stockholm/33/2015 EPI585306
L40I
 A/California/23/2015 EPI587829
 A/Stockholm/36/2015 EPI585330
I365T
86
 A/Philippines/7/2015 EPI636144
 A/Finland/491/2015 EPI598652
95
 A/Hawaii/09/2015 EPI587884
I117M
 A/Bulgaria/456/2015 EPI589498
N386K
 A/Hong Kong/7561/2014 EPI565047
88
 A/Victoria/500/2015 EPI629941
I34V
I321V
K432E
 A/Iraq/478/2015 EPI598657
85
 A/Nepal/2582B/2014 EPI574053
 A/WAKAYAMA/153/2013 EPI497618
 A/Nepal/679A/2014 APR EPI574049
 A/Jilin-Tiexi/SWL1189/2015 EPI586349
90
 A/Nepal/0448/2014 EPI540089
D416N
D451G
E47G, S82P, N397K
 A/Stockholm/21/2015 EPI582111
N200S
N44S
V241I N369K
 A/Hong Kong/8111529/2015 EPI574458
99
99
 A/Cambodia/Y1218309/2014 EPI565258
91
 A/Nepal/0530/2014 EPI540105
 A/Nepal/0506/2014 EPI540097
M19I
92
 A/Ghana/DILI-0620/2014 EPI541038
N248D
 A/Cameroon/13V-3234/2013 NIMR
94
 A/SAPPORO/163/2011 EPI334513
 A/YAMAGATA/752/2009 EPI280328
V106I
 A/Brisbane/10/2010
 A/NARITA/1/2009 EPI179481
 A/California/07/2009 X-179A
 A/California/07/2009
0.002
Suppl Fig. S1 Nakamura et al.
