# Supplementary material for: Characterization of influenza A(H1N1)pdm09 viruses isolated from Nepalese and Indian outbreak patients in early 2015
Source: Influenza Other Respir Viruses. 2017 Aug 9;11(5):399–403. doi: 10.1111/irv.12469 (PMC5596518; doi:10.1111/irv.12469)
Supplement: Supplementary file 2 [file IRV-11-399-s002.pptx]

## Slide 1
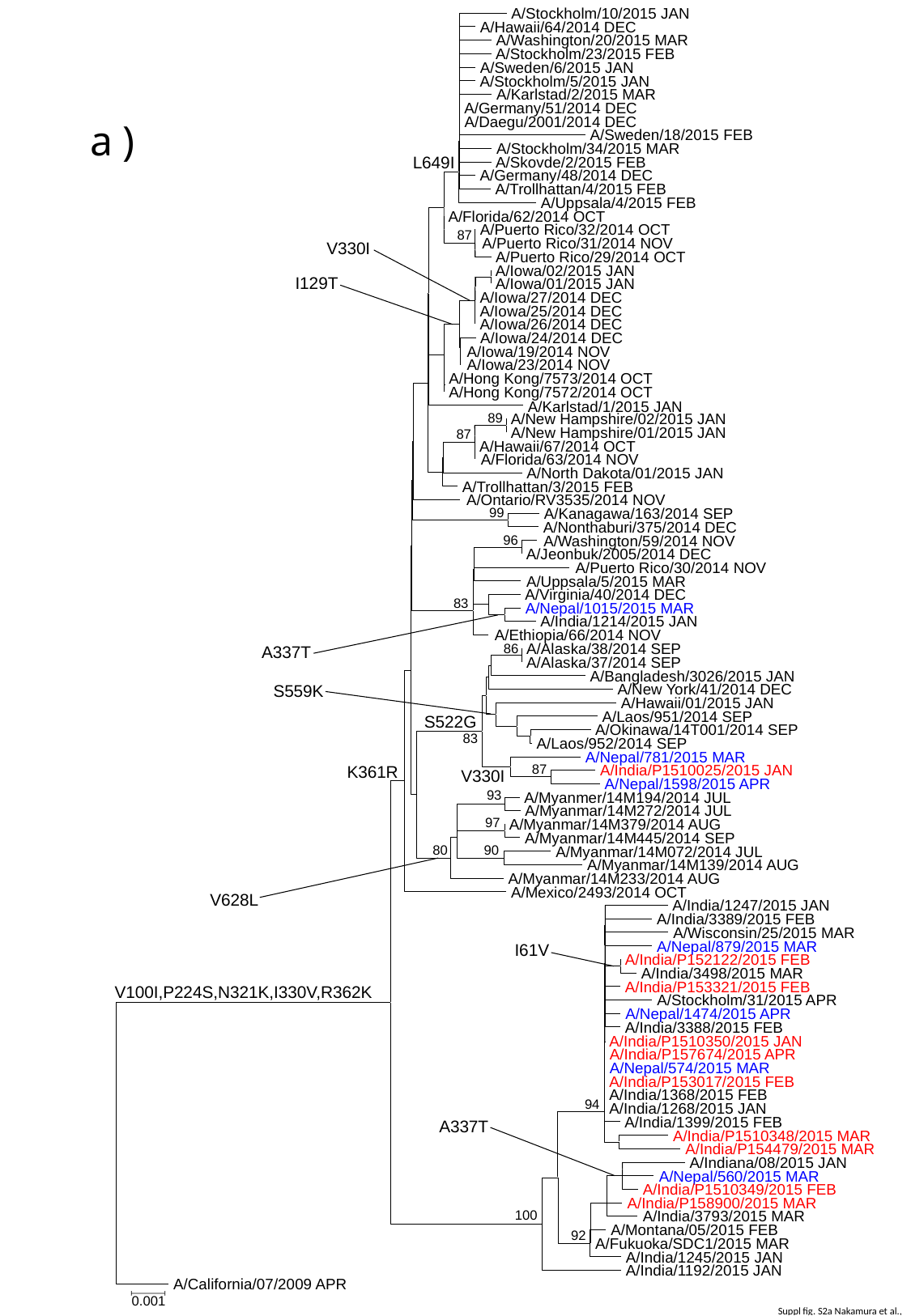

A/Stockholm/10/2015 JAN
 A/Hawaii/64/2014 DEC
 A/Washington/20/2015 MAR
 A/Stockholm/23/2015 FEB
 A/Sweden/6/2015 JAN
 A/Stockholm/5/2015 JAN
 A/Karlstad/2/2015 MAR
 A/Germany/51/2014 DEC
 A/Daegu/2001/2014 DEC
 A/Sweden/18/2015 FEB
 A/Stockholm/34/2015 MAR
 A/Skovde/2/2015 FEB
 A/Germany/48/2014 DEC
 A/Trollhattan/4/2015 FEB
 A/Uppsala/4/2015 FEB
 A/Florida/62/2014 OCT
 A/Puerto Rico/32/2014 OCT
 A/Puerto Rico/31/2014 NOV
 A/Puerto Rico/29/2014 OCT
 A/Iowa/02/2015 JAN
 A/Iowa/01/2015 JAN
 A/Iowa/27/2014 DEC
 A/Iowa/25/2014 DEC
 A/Iowa/26/2014 DEC
 A/Iowa/24/2014 DEC
 A/Iowa/19/2014 NOV
 A/Iowa/23/2014 NOV
 A/Hong Kong/7573/2014 OCT
 A/Hong Kong/7572/2014 OCT
 A/Karlstad/1/2015 JAN
 A/New Hampshire/02/2015 JAN
 A/New Hampshire/01/2015 JAN
 A/Hawaii/67/2014 OCT
 A/Florida/63/2014 NOV
 A/North Dakota/01/2015 JAN
 A/Trollhattan/3/2015 FEB
 A/Ontario/RV3535/2014 NOV
 A/Kanagawa/163/2014 SEP
 A/Nonthaburi/375/2014 DEC
 A/Washington/59/2014 NOV
 A/Jeonbuk/2005/2014 DEC
 A/Puerto Rico/30/2014 NOV
 A/Uppsala/5/2015 MAR
 A/Virginia/40/2014 DEC
 A/Nepal/1015/2015 MAR
 A/India/1214/2015 JAN
 A/Ethiopia/66/2014 NOV
 A/Alaska/38/2014 SEP
 A/Alaska/37/2014 SEP
 A/Bangladesh/3026/2015 JAN
 A/New York/41/2014 DEC
 A/Hawaii/01/2015 JAN
 A/Laos/951/2014 SEP
 A/Okinawa/14T001/2014 SEP
 A/Laos/952/2014 SEP
 A/Nepal/781/2015 MAR
 A/India/P1510025/2015 JAN
 A/Nepal/1598/2015 APR
 A/Myanmer/14M194/2014 JUL
 A/Myanmar/14M272/2014 JUL
 A/Myanmar/14M379/2014 AUG
 A/Myanmar/14M445/2014 SEP
 A/Myanmar/14M072/2014 JUL
 A/Myanmar/14M139/2014 AUG
 A/Myanmar/14M233/2014 AUG
 A/Mexico/2493/2014 OCT
 A/India/1247/2015 JAN
 A/India/3389/2015 FEB
 A/Wisconsin/25/2015 MAR
 A/Nepal/879/2015 MAR
 A/India/P152122/2015 FEB
 A/India/3498/2015 MAR
 A/India/P153321/2015 FEB
 A/Stockholm/31/2015 APR
 A/Nepal/1474/2015 APR
 A/India/3388/2015 FEB
 A/India/P1510350/2015 JAN
 A/India/P157674/2015 APR
 A/Nepal/574/2015 MAR
 A/India/P153017/2015 FEB
 A/India/1368/2015 FEB
 A/India/1268/2015 JAN
 A/India/1399/2015 FEB
 A/India/P1510348/2015 MAR
 A/India/P154479/2015 MAR
 A/Indiana/08/2015 JAN
 A/Nepal/560/2015 MAR
 A/India/P1510349/2015 FEB
 A/India/P158900/2015 MAR
 A/India/3793/2015 MAR
 A/Montana/05/2015 FEB
 A/Fukuoka/SDC1/2015 MAR
 A/India/1245/2015 JAN
 A/India/1192/2015 JAN
 A/California/07/2009 APR
0.001
L649I
V330I
I129T
A337T
S559K
S522G
K361R
V330I
V628L
I61V
V100I,P224S,N321K,I330V,R362K
A337T
a )
87
89
87
99
96
83
86
83
87
93
97
80
90
94
100
92
Suppl fig. S2a Nakamura et al.,

## Slide 2
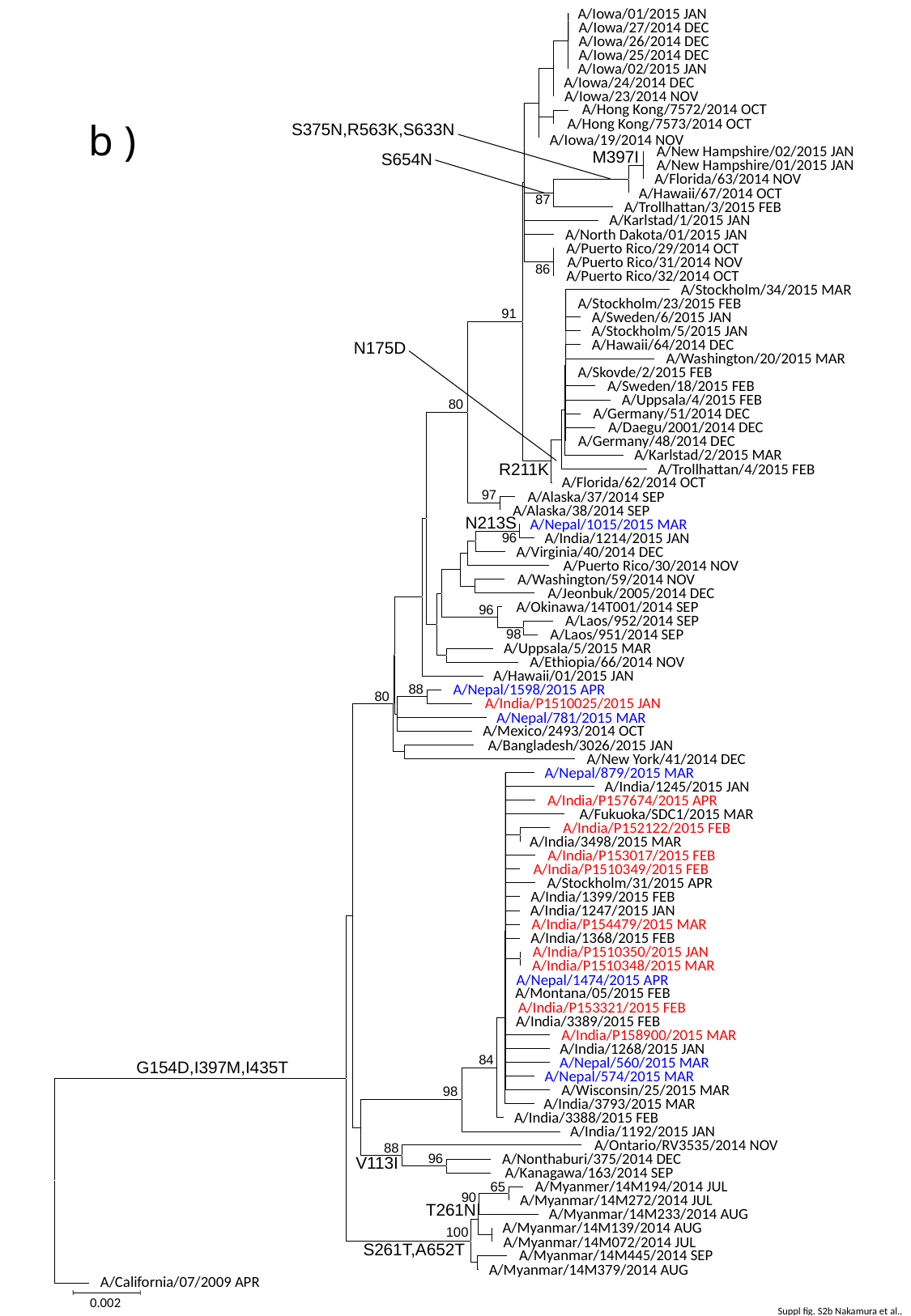

A/Iowa/01/2015 JAN
 A/Iowa/27/2014 DEC
 A/Iowa/26/2014 DEC
 A/Iowa/25/2014 DEC
 A/Iowa/02/2015 JAN
 A/Iowa/24/2014 DEC
 A/Iowa/23/2014 NOV
 A/Hong Kong/7572/2014 OCT
 A/Hong Kong/7573/2014 OCT
 A/Iowa/19/2014 NOV
 A/New Hampshire/02/2015 JAN
 A/New Hampshire/01/2015 JAN
 A/Florida/63/2014 NOV
 A/Hawaii/67/2014 OCT
 A/Trollhattan/3/2015 FEB
 A/Karlstad/1/2015 JAN
 A/North Dakota/01/2015 JAN
 A/Puerto Rico/29/2014 OCT
 A/Puerto Rico/31/2014 NOV
 A/Puerto Rico/32/2014 OCT
 A/Stockholm/34/2015 MAR
 A/Stockholm/23/2015 FEB
 A/Sweden/6/2015 JAN
 A/Stockholm/5/2015 JAN
 A/Hawaii/64/2014 DEC
 A/Washington/20/2015 MAR
 A/Skovde/2/2015 FEB
 A/Sweden/18/2015 FEB
 A/Uppsala/4/2015 FEB
 A/Germany/51/2014 DEC
 A/Daegu/2001/2014 DEC
 A/Germany/48/2014 DEC
 A/Karlstad/2/2015 MAR
 A/Trollhattan/4/2015 FEB
 A/Florida/62/2014 OCT
 A/Alaska/37/2014 SEP
 A/Alaska/38/2014 SEP
 A/Nepal/1015/2015 MAR
 A/India/1214/2015 JAN
 A/Virginia/40/2014 DEC
 A/Puerto Rico/30/2014 NOV
 A/Washington/59/2014 NOV
 A/Jeonbuk/2005/2014 DEC
 A/Okinawa/14T001/2014 SEP
 A/Laos/952/2014 SEP
 A/Laos/951/2014 SEP
 A/Uppsala/5/2015 MAR
 A/Ethiopia/66/2014 NOV
 A/Hawaii/01/2015 JAN
 A/Nepal/1598/2015 APR
 A/India/P1510025/2015 JAN
 A/Nepal/781/2015 MAR
 A/Mexico/2493/2014 OCT
 A/Bangladesh/3026/2015 JAN
 A/New York/41/2014 DEC
 A/Nepal/879/2015 MAR
 A/India/1245/2015 JAN
 A/India/P157674/2015 APR
 A/Fukuoka/SDC1/2015 MAR
 A/India/P152122/2015 FEB
 A/India/3498/2015 MAR
 A/India/P153017/2015 FEB
 A/India/P1510349/2015 FEB
 A/Stockholm/31/2015 APR
 A/India/1399/2015 FEB
 A/India/1247/2015 JAN
 A/India/P154479/2015 MAR
 A/India/1368/2015 FEB
 A/India/P1510350/2015 JAN
 A/India/P1510348/2015 MAR
 A/Nepal/1474/2015 APR
 A/Montana/05/2015 FEB
 A/India/P153321/2015 FEB
 A/India/3389/2015 FEB
 A/India/P158900/2015 MAR
 A/India/1268/2015 JAN
 A/Nepal/560/2015 MAR
 A/Nepal/574/2015 MAR
 A/Wisconsin/25/2015 MAR
 A/India/3793/2015 MAR
 A/India/3388/2015 FEB
 A/India/1192/2015 JAN
 A/Ontario/RV3535/2014 NOV
 A/Nonthaburi/375/2014 DEC
 A/Kanagawa/163/2014 SEP
 A/Myanmer/14M194/2014 JUL
 A/Myanmar/14M272/2014 JUL
 A/Myanmar/14M233/2014 AUG
 A/Myanmar/14M139/2014 AUG
 A/Myanmar/14M072/2014 JUL
 A/Myanmar/14M445/2014 SEP
 A/Myanmar/14M379/2014 AUG
 A/California/07/2009 APR
0.002
S375N,R563K,S633N
M397I
S654N
N175D
R211K
N213S
G154D,I397M,I435T
V113I
T261N
S261T,A652T
b )
87
86
91
80
97
96
96
98
88
80
84
98
88
96
65
90
100
Suppl fig. S2b Nakamura et al.,

## Slide 3
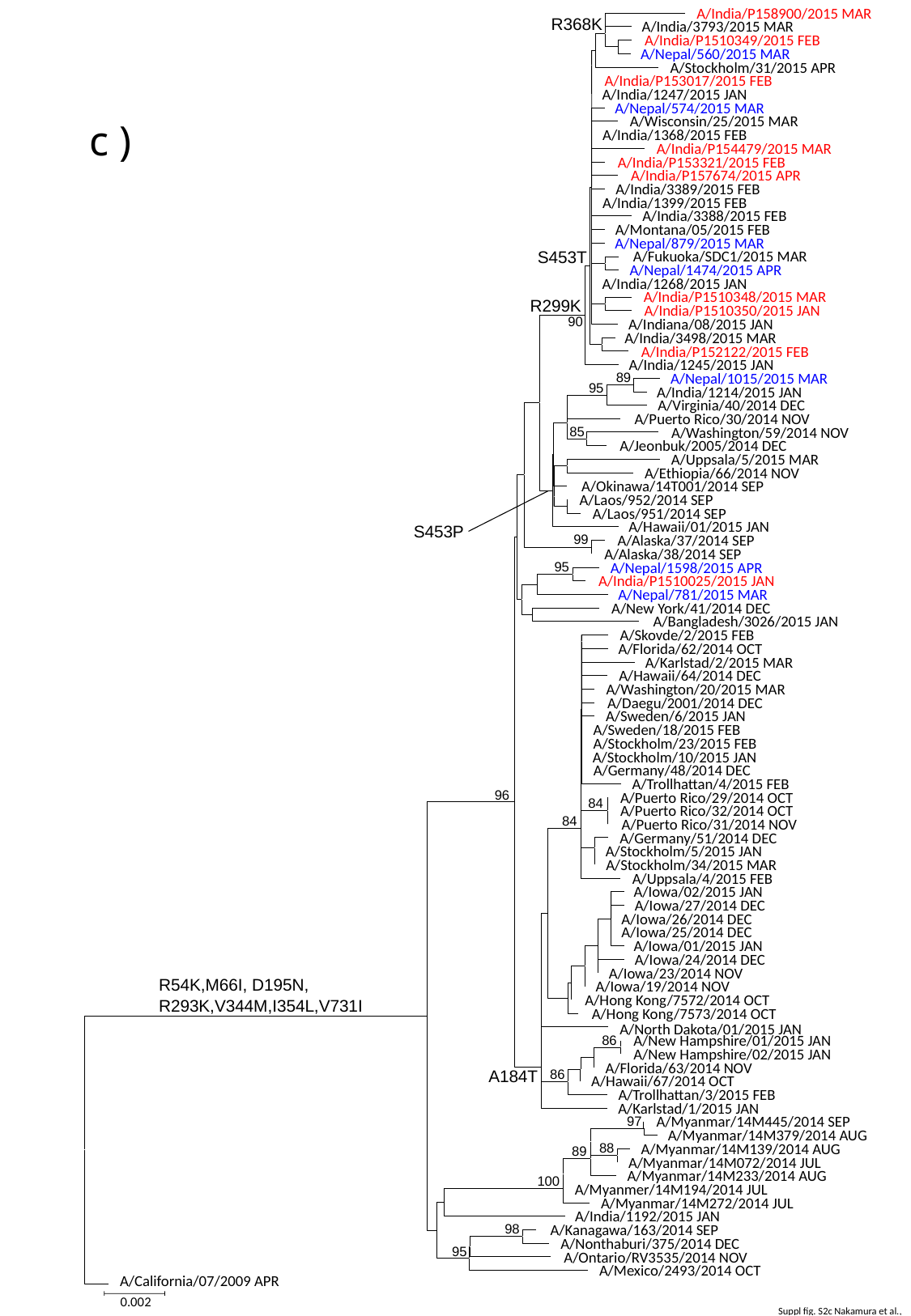

A/India/P158900/2015 MAR
 A/India/3793/2015 MAR
 A/India/P1510349/2015 FEB
 A/Nepal/560/2015 MAR
 A/Stockholm/31/2015 APR
 A/India/P153017/2015 FEB
 A/India/1247/2015 JAN
 A/Nepal/574/2015 MAR
 A/Wisconsin/25/2015 MAR
 A/India/1368/2015 FEB
 A/India/P154479/2015 MAR
 A/India/P153321/2015 FEB
 A/India/P157674/2015 APR
 A/India/3389/2015 FEB
 A/India/1399/2015 FEB
 A/India/3388/2015 FEB
 A/Montana/05/2015 FEB
 A/Nepal/879/2015 MAR
 A/Fukuoka/SDC1/2015 MAR
 A/Nepal/1474/2015 APR
 A/India/1268/2015 JAN
 A/India/P1510348/2015 MAR
 A/India/P1510350/2015 JAN
 A/Indiana/08/2015 JAN
 A/India/3498/2015 MAR
 A/India/P152122/2015 FEB
 A/India/1245/2015 JAN
 A/Nepal/1015/2015 MAR
 A/India/1214/2015 JAN
 A/Virginia/40/2014 DEC
 A/Puerto Rico/30/2014 NOV
 A/Washington/59/2014 NOV
 A/Jeonbuk/2005/2014 DEC
 A/Uppsala/5/2015 MAR
 A/Ethiopia/66/2014 NOV
 A/Okinawa/14T001/2014 SEP
 A/Laos/952/2014 SEP
 A/Laos/951/2014 SEP
 A/Hawaii/01/2015 JAN
 A/Alaska/37/2014 SEP
 A/Alaska/38/2014 SEP
 A/Nepal/1598/2015 APR
 A/India/P1510025/2015 JAN
 A/Nepal/781/2015 MAR
 A/New York/41/2014 DEC
 A/Bangladesh/3026/2015 JAN
 A/Skovde/2/2015 FEB
 A/Florida/62/2014 OCT
 A/Karlstad/2/2015 MAR
 A/Hawaii/64/2014 DEC
 A/Washington/20/2015 MAR
 A/Daegu/2001/2014 DEC
 A/Sweden/6/2015 JAN
 A/Sweden/18/2015 FEB
 A/Stockholm/23/2015 FEB
 A/Stockholm/10/2015 JAN
 A/Germany/48/2014 DEC
 A/Trollhattan/4/2015 FEB
 A/Puerto Rico/29/2014 OCT
 A/Puerto Rico/32/2014 OCT
 A/Puerto Rico/31/2014 NOV
 A/Germany/51/2014 DEC
 A/Stockholm/5/2015 JAN
 A/Stockholm/34/2015 MAR
 A/Uppsala/4/2015 FEB
 A/Iowa/02/2015 JAN
 A/Iowa/27/2014 DEC
 A/Iowa/26/2014 DEC
 A/Iowa/25/2014 DEC
 A/Iowa/01/2015 JAN
 A/Iowa/24/2014 DEC
 A/Iowa/23/2014 NOV
 A/Iowa/19/2014 NOV
 A/Hong Kong/7572/2014 OCT
 A/Hong Kong/7573/2014 OCT
 A/North Dakota/01/2015 JAN
 A/New Hampshire/01/2015 JAN
 A/New Hampshire/02/2015 JAN
 A/Florida/63/2014 NOV
 A/Hawaii/67/2014 OCT
 A/Trollhattan/3/2015 FEB
 A/Karlstad/1/2015 JAN
 A/Myanmar/14M445/2014 SEP
 A/Myanmar/14M379/2014 AUG
 A/Myanmar/14M139/2014 AUG
 A/Myanmar/14M072/2014 JUL
 A/Myanmar/14M233/2014 AUG
 A/Myanmer/14M194/2014 JUL
 A/Myanmar/14M272/2014 JUL
 A/India/1192/2015 JAN
 A/Kanagawa/163/2014 SEP
 A/Nonthaburi/375/2014 DEC
 A/Ontario/RV3535/2014 NOV
 A/Mexico/2493/2014 OCT
 A/California/07/2009 APR
0.002
R368K
c )
S453T
R299K
90
89
95
85
S453P
99
95
96
84
84
R54K,M66I, D195N,
R293K,V344M,I354L,V731I
86
A184T
86
97
88
89
100
98
95
Suppl fig. S2c Nakamura et al.,

## Slide 4
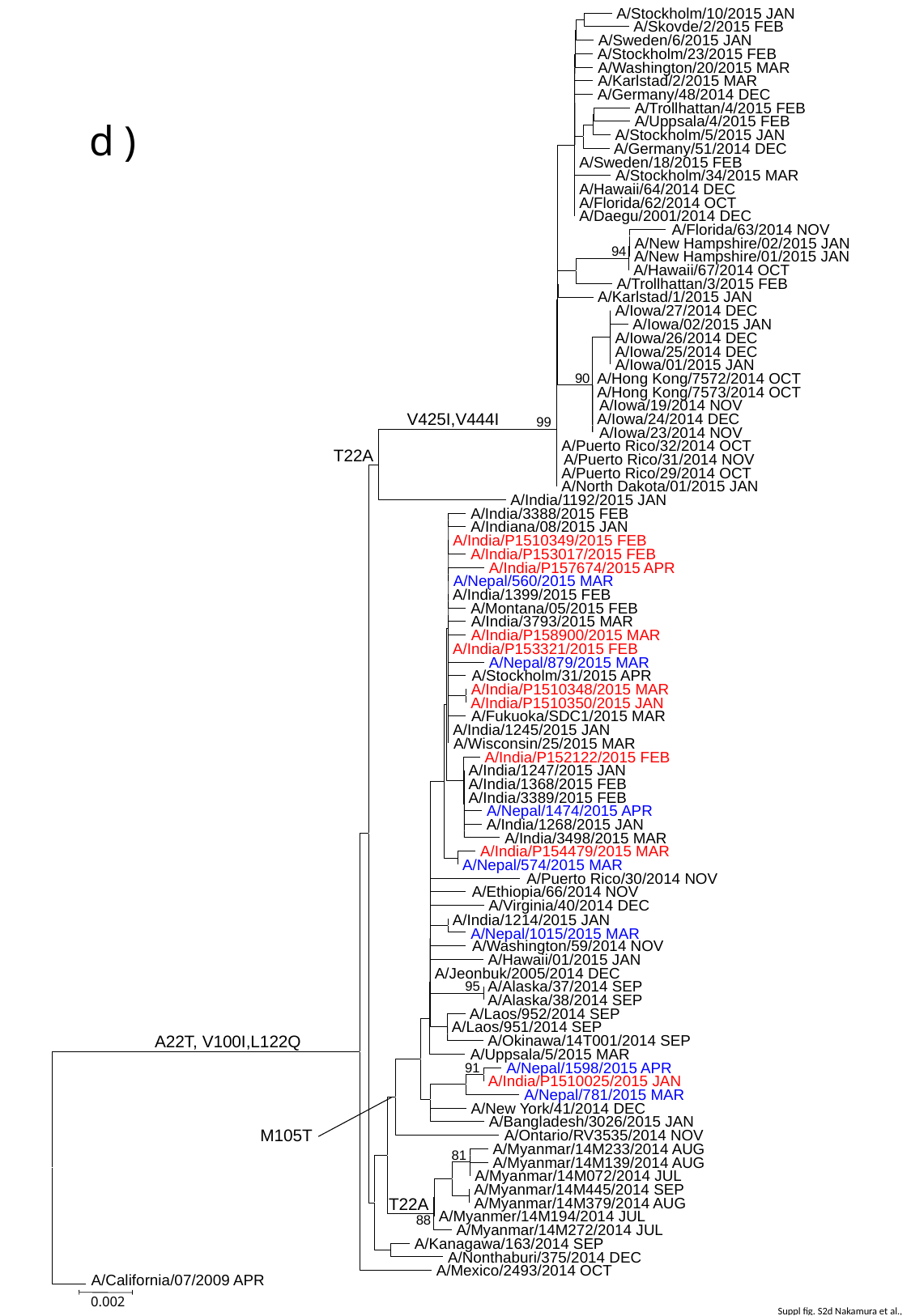

A/Stockholm/10/2015 JAN
 A/Skovde/2/2015 FEB
 A/Sweden/6/2015 JAN
 A/Stockholm/23/2015 FEB
 A/Washington/20/2015 MAR
 A/Karlstad/2/2015 MAR
 A/Germany/48/2014 DEC
 A/Trollhattan/4/2015 FEB
 A/Uppsala/4/2015 FEB
 A/Stockholm/5/2015 JAN
 A/Germany/51/2014 DEC
 A/Sweden/18/2015 FEB
 A/Stockholm/34/2015 MAR
 A/Hawaii/64/2014 DEC
 A/Florida/62/2014 OCT
 A/Daegu/2001/2014 DEC
 A/Florida/63/2014 NOV
 A/New Hampshire/02/2015 JAN
 A/New Hampshire/01/2015 JAN
 A/Hawaii/67/2014 OCT
 A/Trollhattan/3/2015 FEB
 A/Karlstad/1/2015 JAN
 A/Iowa/27/2014 DEC
 A/Iowa/02/2015 JAN
 A/Iowa/26/2014 DEC
 A/Iowa/25/2014 DEC
 A/Iowa/01/2015 JAN
 A/Hong Kong/7572/2014 OCT
 A/Hong Kong/7573/2014 OCT
 A/Iowa/19/2014 NOV
 A/Iowa/24/2014 DEC
 A/Iowa/23/2014 NOV
 A/Puerto Rico/32/2014 OCT
 A/Puerto Rico/31/2014 NOV
 A/Puerto Rico/29/2014 OCT
 A/North Dakota/01/2015 JAN
 A/India/1192/2015 JAN
 A/India/3388/2015 FEB
 A/Indiana/08/2015 JAN
 A/India/P1510349/2015 FEB
 A/India/P153017/2015 FEB
 A/India/P157674/2015 APR
 A/Nepal/560/2015 MAR
 A/India/1399/2015 FEB
 A/Montana/05/2015 FEB
 A/India/3793/2015 MAR
 A/India/P158900/2015 MAR
 A/India/P153321/2015 FEB
 A/Nepal/879/2015 MAR
 A/Stockholm/31/2015 APR
 A/India/P1510348/2015 MAR
 A/India/P1510350/2015 JAN
 A/Fukuoka/SDC1/2015 MAR
 A/India/1245/2015 JAN
 A/Wisconsin/25/2015 MAR
 A/India/P152122/2015 FEB
 A/India/1247/2015 JAN
 A/India/1368/2015 FEB
 A/India/3389/2015 FEB
 A/Nepal/1474/2015 APR
 A/India/1268/2015 JAN
 A/India/3498/2015 MAR
 A/India/P154479/2015 MAR
 A/Nepal/574/2015 MAR
 A/Puerto Rico/30/2014 NOV
 A/Ethiopia/66/2014 NOV
 A/Virginia/40/2014 DEC
 A/India/1214/2015 JAN
 A/Nepal/1015/2015 MAR
 A/Washington/59/2014 NOV
 A/Hawaii/01/2015 JAN
 A/Jeonbuk/2005/2014 DEC
 A/Alaska/37/2014 SEP
 A/Alaska/38/2014 SEP
 A/Laos/952/2014 SEP
 A/Laos/951/2014 SEP
 A/Okinawa/14T001/2014 SEP
 A/Uppsala/5/2015 MAR
 A/Nepal/1598/2015 APR
 A/India/P1510025/2015 JAN
 A/Nepal/781/2015 MAR
 A/New York/41/2014 DEC
 A/Bangladesh/3026/2015 JAN
 A/Ontario/RV3535/2014 NOV
 A/Myanmar/14M233/2014 AUG
 A/Myanmar/14M139/2014 AUG
 A/Myanmar/14M072/2014 JUL
 A/Myanmar/14M445/2014 SEP
 A/Myanmar/14M379/2014 AUG
 A/Myanmer/14M194/2014 JUL
 A/Myanmar/14M272/2014 JUL
 A/Kanagawa/163/2014 SEP
 A/Nonthaburi/375/2014 DEC
 A/Mexico/2493/2014 OCT
 A/California/07/2009 APR
0.002
d )
94
90
V425I,V444I
99
T22A
95
A22T, V100I,L122Q
91
M105T
81
T22A
88
Suppl fig. S2d Nakamura et al.,

## Slide 5
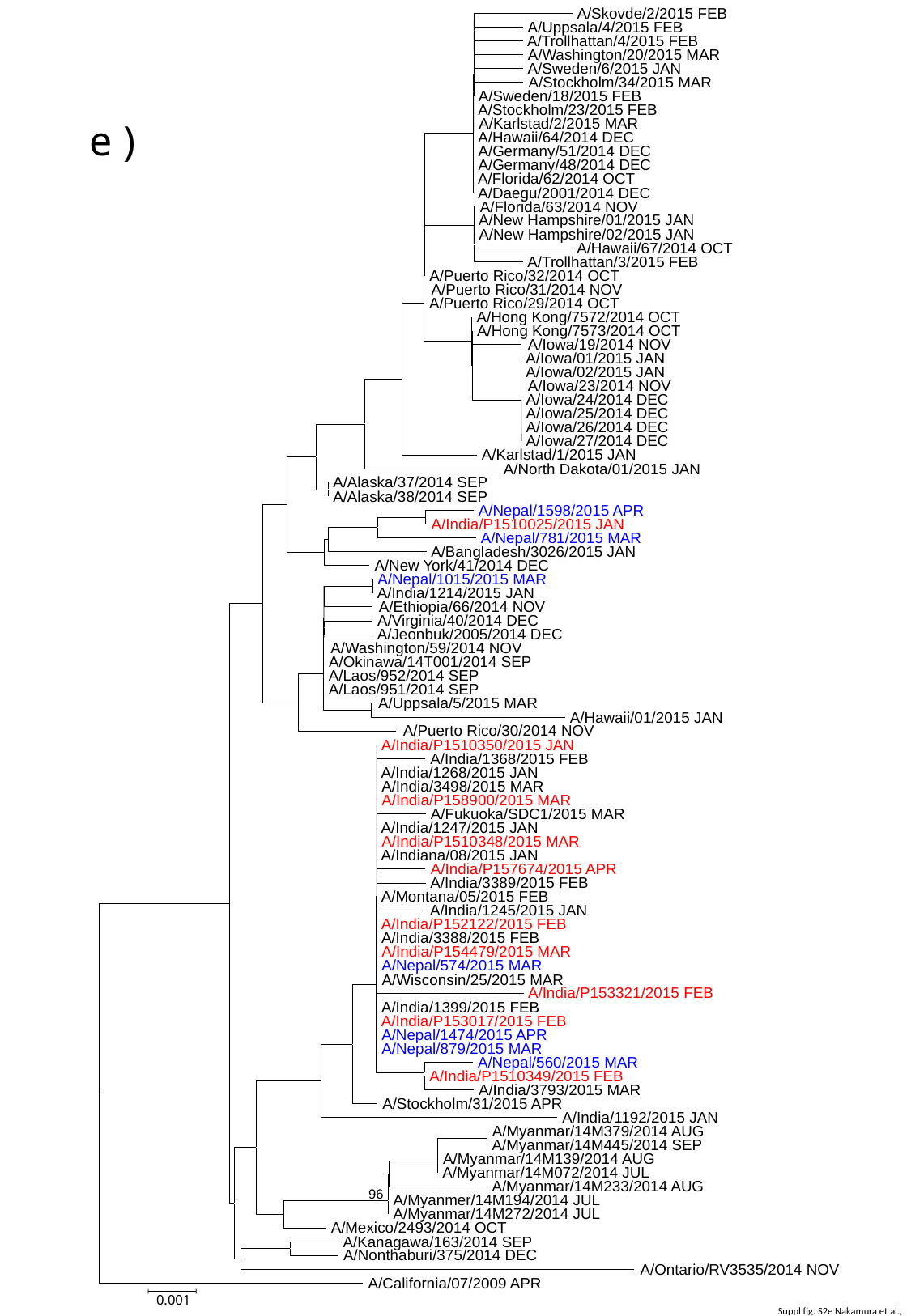

A/Skovde/2/2015 FEB
 A/Uppsala/4/2015 FEB
 A/Trollhattan/4/2015 FEB
 A/Washington/20/2015 MAR
 A/Sweden/6/2015 JAN
 A/Stockholm/34/2015 MAR
 A/Sweden/18/2015 FEB
 A/Stockholm/23/2015 FEB
 A/Karlstad/2/2015 MAR
 A/Hawaii/64/2014 DEC
 A/Germany/51/2014 DEC
 A/Germany/48/2014 DEC
 A/Florida/62/2014 OCT
 A/Daegu/2001/2014 DEC
 A/Florida/63/2014 NOV
 A/New Hampshire/01/2015 JAN
 A/New Hampshire/02/2015 JAN
 A/Hawaii/67/2014 OCT
 A/Trollhattan/3/2015 FEB
 A/Puerto Rico/32/2014 OCT
 A/Puerto Rico/31/2014 NOV
 A/Puerto Rico/29/2014 OCT
 A/Hong Kong/7572/2014 OCT
 A/Hong Kong/7573/2014 OCT
 A/Iowa/19/2014 NOV
 A/Iowa/01/2015 JAN
 A/Iowa/02/2015 JAN
 A/Iowa/23/2014 NOV
 A/Iowa/24/2014 DEC
 A/Iowa/25/2014 DEC
 A/Iowa/26/2014 DEC
 A/Iowa/27/2014 DEC
 A/Karlstad/1/2015 JAN
 A/North Dakota/01/2015 JAN
 A/Alaska/37/2014 SEP
 A/Alaska/38/2014 SEP
 A/Nepal/1598/2015 APR
 A/India/P1510025/2015 JAN
 A/Nepal/781/2015 MAR
 A/Bangladesh/3026/2015 JAN
 A/New York/41/2014 DEC
 A/Nepal/1015/2015 MAR
 A/India/1214/2015 JAN
 A/Ethiopia/66/2014 NOV
 A/Virginia/40/2014 DEC
 A/Jeonbuk/2005/2014 DEC
 A/Washington/59/2014 NOV
 A/Okinawa/14T001/2014 SEP
 A/Laos/952/2014 SEP
 A/Laos/951/2014 SEP
 A/Uppsala/5/2015 MAR
 A/Hawaii/01/2015 JAN
 A/Puerto Rico/30/2014 NOV
 A/India/P1510350/2015 JAN
 A/India/1368/2015 FEB
 A/India/1268/2015 JAN
 A/India/3498/2015 MAR
 A/India/P158900/2015 MAR
 A/Fukuoka/SDC1/2015 MAR
 A/India/1247/2015 JAN
 A/India/P1510348/2015 MAR
 A/Indiana/08/2015 JAN
 A/India/P157674/2015 APR
 A/India/3389/2015 FEB
 A/Montana/05/2015 FEB
 A/India/1245/2015 JAN
 A/India/P152122/2015 FEB
 A/India/3388/2015 FEB
 A/India/P154479/2015 MAR
 A/Nepal/574/2015 MAR
 A/Wisconsin/25/2015 MAR
 A/India/P153321/2015 FEB
 A/India/1399/2015 FEB
 A/India/P153017/2015 FEB
 A/Nepal/1474/2015 APR
 A/Nepal/879/2015 MAR
 A/Nepal/560/2015 MAR
 A/India/P1510349/2015 FEB
 A/India/3793/2015 MAR
 A/Stockholm/31/2015 APR
 A/India/1192/2015 JAN
 A/Myanmar/14M379/2014 AUG
 A/Myanmar/14M445/2014 SEP
 A/Myanmar/14M139/2014 AUG
 A/Myanmar/14M072/2014 JUL
 A/Myanmar/14M233/2014 AUG
 A/Myanmer/14M194/2014 JUL
 A/Myanmar/14M272/2014 JUL
 A/Mexico/2493/2014 OCT
 A/Kanagawa/163/2014 SEP
 A/Nonthaburi/375/2014 DEC
 A/Ontario/RV3535/2014 NOV
 A/California/07/2009 APR
0.001
e )
96
Suppl fig. S2e Nakamura et al.,

## Slide 6
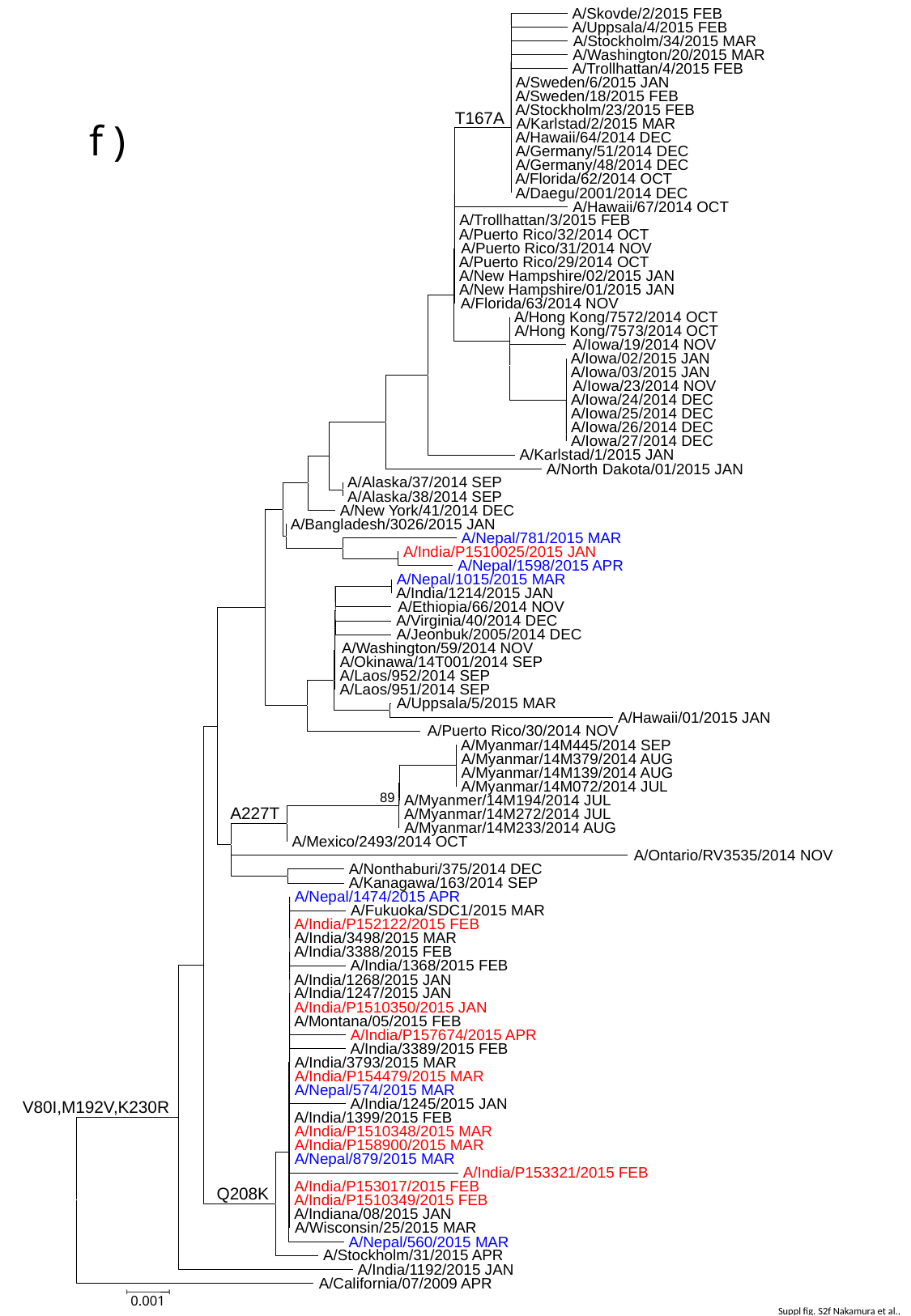

A/Skovde/2/2015 FEB
 A/Uppsala/4/2015 FEB
 A/Stockholm/34/2015 MAR
 A/Washington/20/2015 MAR
 A/Trollhattan/4/2015 FEB
 A/Sweden/6/2015 JAN
 A/Sweden/18/2015 FEB
 A/Stockholm/23/2015 FEB
 A/Karlstad/2/2015 MAR
 A/Hawaii/64/2014 DEC
 A/Germany/51/2014 DEC
 A/Germany/48/2014 DEC
 A/Florida/62/2014 OCT
 A/Daegu/2001/2014 DEC
 A/Hawaii/67/2014 OCT
 A/Trollhattan/3/2015 FEB
 A/Puerto Rico/32/2014 OCT
 A/Puerto Rico/31/2014 NOV
 A/Puerto Rico/29/2014 OCT
 A/New Hampshire/02/2015 JAN
 A/New Hampshire/01/2015 JAN
 A/Florida/63/2014 NOV
 A/Hong Kong/7572/2014 OCT
 A/Hong Kong/7573/2014 OCT
 A/Iowa/19/2014 NOV
 A/Iowa/02/2015 JAN
 A/Iowa/03/2015 JAN
 A/Iowa/23/2014 NOV
 A/Iowa/24/2014 DEC
 A/Iowa/25/2014 DEC
 A/Iowa/26/2014 DEC
 A/Iowa/27/2014 DEC
 A/Karlstad/1/2015 JAN
 A/North Dakota/01/2015 JAN
 A/Alaska/37/2014 SEP
 A/Alaska/38/2014 SEP
 A/New York/41/2014 DEC
 A/Bangladesh/3026/2015 JAN
 A/Nepal/781/2015 MAR
 A/India/P1510025/2015 JAN
 A/Nepal/1598/2015 APR
 A/Nepal/1015/2015 MAR
 A/India/1214/2015 JAN
 A/Ethiopia/66/2014 NOV
 A/Virginia/40/2014 DEC
 A/Jeonbuk/2005/2014 DEC
 A/Washington/59/2014 NOV
 A/Okinawa/14T001/2014 SEP
 A/Laos/952/2014 SEP
 A/Laos/951/2014 SEP
 A/Uppsala/5/2015 MAR
 A/Hawaii/01/2015 JAN
 A/Puerto Rico/30/2014 NOV
 A/Myanmar/14M445/2014 SEP
 A/Myanmar/14M379/2014 AUG
 A/Myanmar/14M139/2014 AUG
 A/Myanmar/14M072/2014 JUL
 A/Myanmer/14M194/2014 JUL
 A/Myanmar/14M272/2014 JUL
 A/Myanmar/14M233/2014 AUG
 A/Mexico/2493/2014 OCT
 A/Ontario/RV3535/2014 NOV
 A/Nonthaburi/375/2014 DEC
 A/Kanagawa/163/2014 SEP
 A/Nepal/1474/2015 APR
 A/Fukuoka/SDC1/2015 MAR
 A/India/P152122/2015 FEB
 A/India/3498/2015 MAR
 A/India/3388/2015 FEB
 A/India/1368/2015 FEB
 A/India/1268/2015 JAN
 A/India/1247/2015 JAN
 A/India/P1510350/2015 JAN
 A/Montana/05/2015 FEB
 A/India/P157674/2015 APR
 A/India/3389/2015 FEB
 A/India/3793/2015 MAR
 A/India/P154479/2015 MAR
 A/Nepal/574/2015 MAR
 A/India/1245/2015 JAN
 A/India/1399/2015 FEB
 A/India/P1510348/2015 MAR
 A/India/P158900/2015 MAR
 A/Nepal/879/2015 MAR
 A/India/P153321/2015 FEB
 A/India/P153017/2015 FEB
 A/India/P1510349/2015 FEB
 A/Indiana/08/2015 JAN
 A/Wisconsin/25/2015 MAR
 A/Nepal/560/2015 MAR
 A/Stockholm/31/2015 APR
 A/India/1192/2015 JAN
 A/California/07/2009 APR
0.001
T167A
f )
89
A227T
V80I,M192V,K230R
Q208K
Suppl fig. S2f Nakamura et al.,

## Slide 7
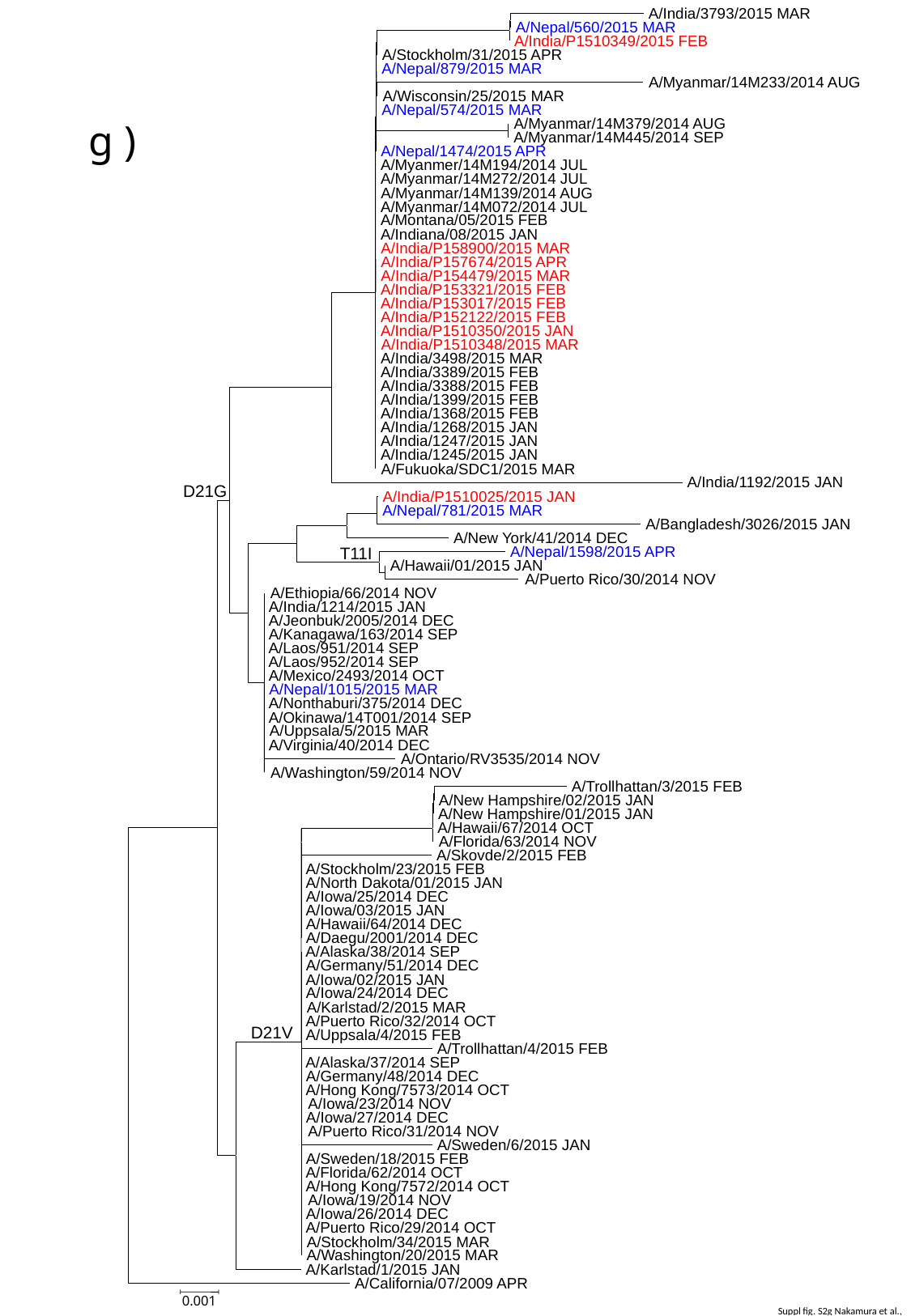

A/India/3793/2015 MAR
 A/Nepal/560/2015 MAR
 A/India/P1510349/2015 FEB
 A/Stockholm/31/2015 APR
 A/Nepal/879/2015 MAR
 A/Myanmar/14M233/2014 AUG
 A/Wisconsin/25/2015 MAR
 A/Nepal/574/2015 MAR
 A/Myanmar/14M379/2014 AUG
 A/Myanmar/14M445/2014 SEP
 A/Nepal/1474/2015 APR
 A/Myanmer/14M194/2014 JUL
 A/Myanmar/14M272/2014 JUL
 A/Myanmar/14M139/2014 AUG
 A/Myanmar/14M072/2014 JUL
 A/Montana/05/2015 FEB
 A/Indiana/08/2015 JAN
 A/India/P158900/2015 MAR
 A/India/P157674/2015 APR
 A/India/P154479/2015 MAR
 A/India/P153321/2015 FEB
 A/India/P153017/2015 FEB
 A/India/P152122/2015 FEB
 A/India/P1510350/2015 JAN
 A/India/P1510348/2015 MAR
 A/India/3498/2015 MAR
 A/India/3389/2015 FEB
 A/India/3388/2015 FEB
 A/India/1399/2015 FEB
 A/India/1368/2015 FEB
 A/India/1268/2015 JAN
 A/India/1247/2015 JAN
 A/India/1245/2015 JAN
 A/Fukuoka/SDC1/2015 MAR
 A/India/1192/2015 JAN
 A/India/P1510025/2015 JAN
 A/Nepal/781/2015 MAR
 A/Bangladesh/3026/2015 JAN
 A/New York/41/2014 DEC
 A/Nepal/1598/2015 APR
 A/Hawaii/01/2015 JAN
 A/Puerto Rico/30/2014 NOV
 A/Ethiopia/66/2014 NOV
 A/India/1214/2015 JAN
 A/Jeonbuk/2005/2014 DEC
 A/Kanagawa/163/2014 SEP
 A/Laos/951/2014 SEP
 A/Laos/952/2014 SEP
 A/Mexico/2493/2014 OCT
 A/Nepal/1015/2015 MAR
 A/Nonthaburi/375/2014 DEC
 A/Okinawa/14T001/2014 SEP
 A/Uppsala/5/2015 MAR
 A/Virginia/40/2014 DEC
 A/Ontario/RV3535/2014 NOV
 A/Washington/59/2014 NOV
 A/Trollhattan/3/2015 FEB
 A/New Hampshire/02/2015 JAN
 A/New Hampshire/01/2015 JAN
 A/Hawaii/67/2014 OCT
 A/Florida/63/2014 NOV
 A/Skovde/2/2015 FEB
 A/Stockholm/23/2015 FEB
 A/North Dakota/01/2015 JAN
 A/Iowa/25/2014 DEC
 A/Iowa/03/2015 JAN
 A/Hawaii/64/2014 DEC
 A/Daegu/2001/2014 DEC
 A/Alaska/38/2014 SEP
 A/Germany/51/2014 DEC
 A/Iowa/02/2015 JAN
 A/Iowa/24/2014 DEC
 A/Karlstad/2/2015 MAR
 A/Puerto Rico/32/2014 OCT
 A/Uppsala/4/2015 FEB
 A/Trollhattan/4/2015 FEB
 A/Alaska/37/2014 SEP
 A/Germany/48/2014 DEC
 A/Hong Kong/7573/2014 OCT
 A/Iowa/23/2014 NOV
 A/Iowa/27/2014 DEC
 A/Puerto Rico/31/2014 NOV
 A/Sweden/6/2015 JAN
 A/Sweden/18/2015 FEB
 A/Florida/62/2014 OCT
 A/Hong Kong/7572/2014 OCT
 A/Iowa/19/2014 NOV
 A/Iowa/26/2014 DEC
 A/Puerto Rico/29/2014 OCT
 A/Stockholm/34/2015 MAR
 A/Washington/20/2015 MAR
 A/Karlstad/1/2015 JAN
 A/California/07/2009 APR
0.001
g )
D21G
T11I
D21V
Suppl fig. S2g Nakamura et al.,

## Slide 8
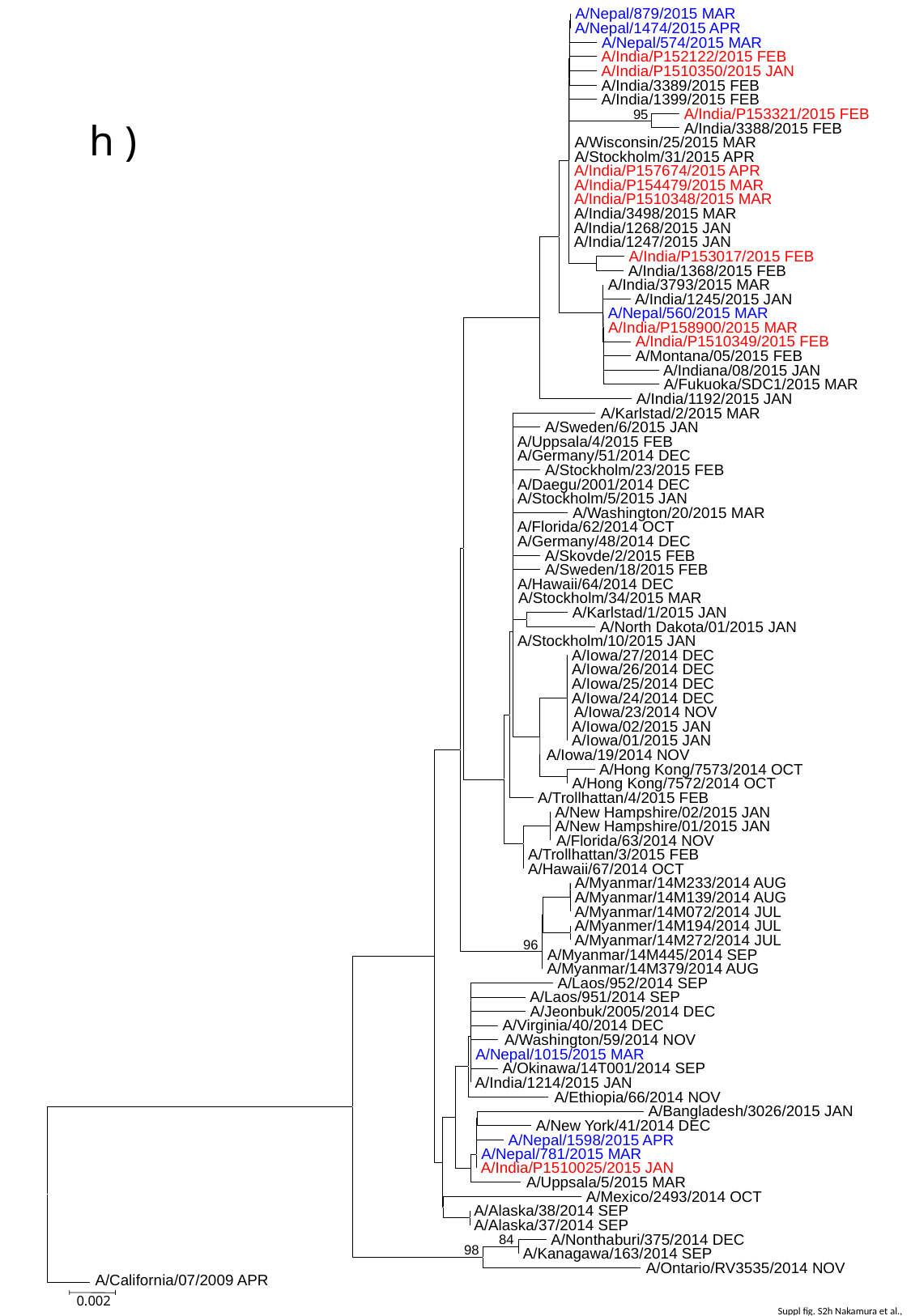

A/Nepal/879/2015 MAR
 A/Nepal/1474/2015 APR
 A/Nepal/574/2015 MAR
 A/India/P152122/2015 FEB
 A/India/P1510350/2015 JAN
 A/India/3389/2015 FEB
 A/India/1399/2015 FEB
 A/India/P153321/2015 FEB
 A/India/3388/2015 FEB
 A/Wisconsin/25/2015 MAR
 A/Stockholm/31/2015 APR
 A/India/P157674/2015 APR
 A/India/P154479/2015 MAR
 A/India/P1510348/2015 MAR
 A/India/3498/2015 MAR
 A/India/1268/2015 JAN
 A/India/1247/2015 JAN
 A/India/P153017/2015 FEB
 A/India/1368/2015 FEB
 A/India/3793/2015 MAR
 A/India/1245/2015 JAN
 A/Nepal/560/2015 MAR
 A/India/P158900/2015 MAR
 A/India/P1510349/2015 FEB
 A/Montana/05/2015 FEB
 A/Indiana/08/2015 JAN
 A/Fukuoka/SDC1/2015 MAR
 A/India/1192/2015 JAN
 A/Karlstad/2/2015 MAR
 A/Sweden/6/2015 JAN
 A/Uppsala/4/2015 FEB
 A/Germany/51/2014 DEC
 A/Stockholm/23/2015 FEB
 A/Daegu/2001/2014 DEC
 A/Stockholm/5/2015 JAN
 A/Washington/20/2015 MAR
 A/Florida/62/2014 OCT
 A/Germany/48/2014 DEC
 A/Skovde/2/2015 FEB
 A/Sweden/18/2015 FEB
 A/Hawaii/64/2014 DEC
 A/Stockholm/34/2015 MAR
 A/Karlstad/1/2015 JAN
 A/North Dakota/01/2015 JAN
 A/Stockholm/10/2015 JAN
 A/Iowa/27/2014 DEC
 A/Iowa/26/2014 DEC
 A/Iowa/25/2014 DEC
 A/Iowa/24/2014 DEC
 A/Iowa/23/2014 NOV
 A/Iowa/02/2015 JAN
 A/Iowa/01/2015 JAN
 A/Iowa/19/2014 NOV
 A/Hong Kong/7573/2014 OCT
 A/Hong Kong/7572/2014 OCT
 A/Trollhattan/4/2015 FEB
 A/New Hampshire/02/2015 JAN
 A/New Hampshire/01/2015 JAN
 A/Florida/63/2014 NOV
 A/Trollhattan/3/2015 FEB
 A/Hawaii/67/2014 OCT
 A/Myanmar/14M233/2014 AUG
 A/Myanmar/14M139/2014 AUG
 A/Myanmar/14M072/2014 JUL
 A/Myanmer/14M194/2014 JUL
 A/Myanmar/14M272/2014 JUL
 A/Myanmar/14M445/2014 SEP
 A/Myanmar/14M379/2014 AUG
 A/Laos/952/2014 SEP
 A/Laos/951/2014 SEP
 A/Jeonbuk/2005/2014 DEC
 A/Virginia/40/2014 DEC
 A/Washington/59/2014 NOV
 A/Nepal/1015/2015 MAR
 A/Okinawa/14T001/2014 SEP
 A/India/1214/2015 JAN
 A/Ethiopia/66/2014 NOV
 A/Bangladesh/3026/2015 JAN
 A/New York/41/2014 DEC
 A/Nepal/1598/2015 APR
 A/Nepal/781/2015 MAR
 A/India/P1510025/2015 JAN
 A/Uppsala/5/2015 MAR
 A/Mexico/2493/2014 OCT
 A/Alaska/38/2014 SEP
 A/Alaska/37/2014 SEP
 A/Nonthaburi/375/2014 DEC
 A/Kanagawa/163/2014 SEP
 A/Ontario/RV3535/2014 NOV
 A/California/07/2009 APR
0.002
95
h )
96
84
98
Suppl fig. S2h Nakamura et al.,

## Slide 9
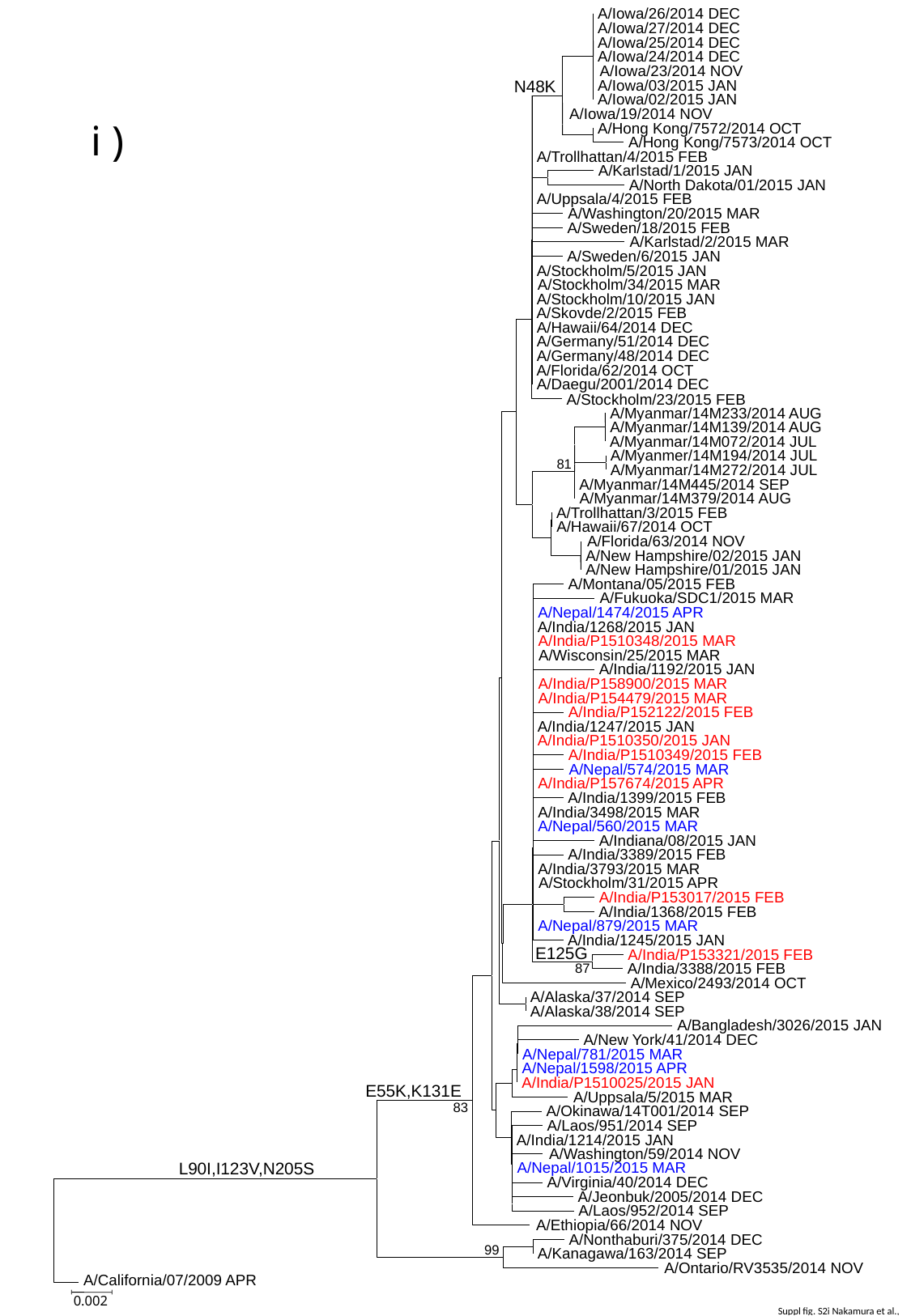

A/Iowa/26/2014 DEC
 A/Iowa/27/2014 DEC
 A/Iowa/25/2014 DEC
 A/Iowa/24/2014 DEC
 A/Iowa/23/2014 NOV
 A/Iowa/03/2015 JAN
 A/Iowa/02/2015 JAN
 A/Iowa/19/2014 NOV
 A/Hong Kong/7572/2014 OCT
 A/Hong Kong/7573/2014 OCT
 A/Trollhattan/4/2015 FEB
 A/Karlstad/1/2015 JAN
 A/North Dakota/01/2015 JAN
 A/Uppsala/4/2015 FEB
 A/Washington/20/2015 MAR
 A/Sweden/18/2015 FEB
 A/Karlstad/2/2015 MAR
 A/Sweden/6/2015 JAN
 A/Stockholm/5/2015 JAN
 A/Stockholm/34/2015 MAR
 A/Stockholm/10/2015 JAN
 A/Skovde/2/2015 FEB
 A/Hawaii/64/2014 DEC
 A/Germany/51/2014 DEC
 A/Germany/48/2014 DEC
 A/Florida/62/2014 OCT
 A/Daegu/2001/2014 DEC
 A/Stockholm/23/2015 FEB
 A/Myanmar/14M233/2014 AUG
 A/Myanmar/14M139/2014 AUG
 A/Myanmar/14M072/2014 JUL
 A/Myanmer/14M194/2014 JUL
 A/Myanmar/14M272/2014 JUL
 A/Myanmar/14M445/2014 SEP
 A/Myanmar/14M379/2014 AUG
 A/Trollhattan/3/2015 FEB
 A/Hawaii/67/2014 OCT
 A/Florida/63/2014 NOV
 A/New Hampshire/02/2015 JAN
 A/New Hampshire/01/2015 JAN
 A/Montana/05/2015 FEB
 A/Fukuoka/SDC1/2015 MAR
 A/Nepal/1474/2015 APR
 A/India/1268/2015 JAN
 A/India/P1510348/2015 MAR
 A/Wisconsin/25/2015 MAR
 A/India/1192/2015 JAN
 A/India/P158900/2015 MAR
 A/India/P154479/2015 MAR
 A/India/P152122/2015 FEB
 A/India/1247/2015 JAN
 A/India/P1510350/2015 JAN
 A/India/P1510349/2015 FEB
 A/Nepal/574/2015 MAR
 A/India/P157674/2015 APR
 A/India/1399/2015 FEB
 A/India/3498/2015 MAR
 A/Nepal/560/2015 MAR
 A/Indiana/08/2015 JAN
 A/India/3389/2015 FEB
 A/India/3793/2015 MAR
 A/Stockholm/31/2015 APR
 A/India/P153017/2015 FEB
 A/India/1368/2015 FEB
 A/Nepal/879/2015 MAR
 A/India/1245/2015 JAN
 A/India/P153321/2015 FEB
 A/India/3388/2015 FEB
 A/Mexico/2493/2014 OCT
 A/Alaska/37/2014 SEP
 A/Alaska/38/2014 SEP
 A/Bangladesh/3026/2015 JAN
 A/New York/41/2014 DEC
 A/Nepal/781/2015 MAR
 A/Nepal/1598/2015 APR
 A/India/P1510025/2015 JAN
 A/Uppsala/5/2015 MAR
 A/Okinawa/14T001/2014 SEP
 A/Laos/951/2014 SEP
 A/India/1214/2015 JAN
 A/Washington/59/2014 NOV
 A/Nepal/1015/2015 MAR
 A/Virginia/40/2014 DEC
 A/Jeonbuk/2005/2014 DEC
 A/Laos/952/2014 SEP
 A/Ethiopia/66/2014 NOV
 A/Nonthaburi/375/2014 DEC
 A/Kanagawa/163/2014 SEP
 A/Ontario/RV3535/2014 NOV
 A/California/07/2009 APR
0.002
N48K
i )
81
E125G
87
E55K,K131E
83
L90I,I123V,N205S
99
Suppl fig. S2i Nakamura et al.,

## Slide 10
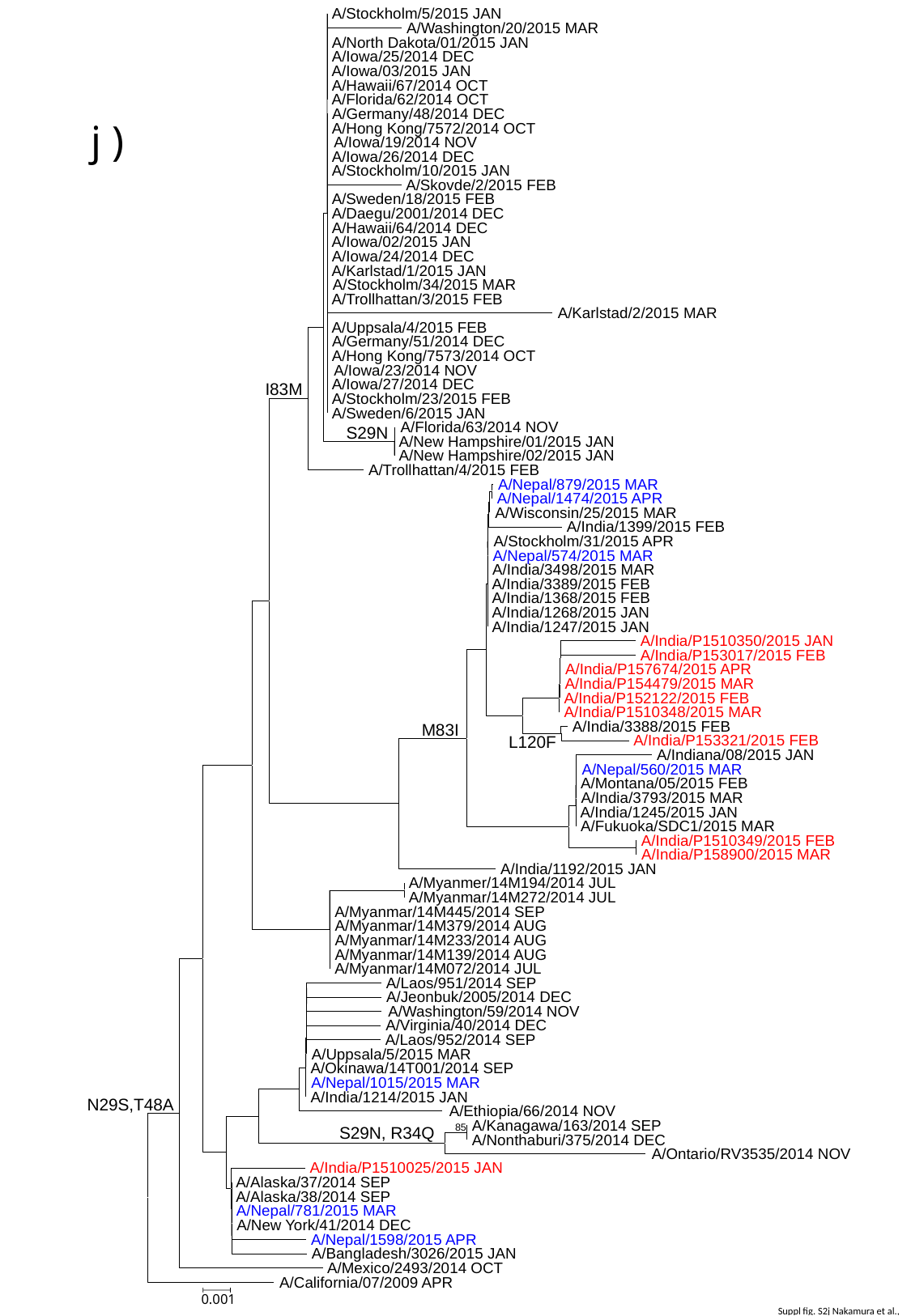

A/Stockholm/5/2015 JAN
 A/Washington/20/2015 MAR
 A/North Dakota/01/2015 JAN
 A/Iowa/25/2014 DEC
 A/Iowa/03/2015 JAN
 A/Hawaii/67/2014 OCT
 A/Florida/62/2014 OCT
 A/Germany/48/2014 DEC
 A/Hong Kong/7572/2014 OCT
 A/Iowa/19/2014 NOV
 A/Iowa/26/2014 DEC
 A/Stockholm/10/2015 JAN
 A/Skovde/2/2015 FEB
 A/Sweden/18/2015 FEB
 A/Daegu/2001/2014 DEC
 A/Hawaii/64/2014 DEC
 A/Iowa/02/2015 JAN
 A/Iowa/24/2014 DEC
 A/Karlstad/1/2015 JAN
 A/Stockholm/34/2015 MAR
 A/Trollhattan/3/2015 FEB
 A/Karlstad/2/2015 MAR
 A/Uppsala/4/2015 FEB
 A/Germany/51/2014 DEC
 A/Hong Kong/7573/2014 OCT
 A/Iowa/23/2014 NOV
 A/Iowa/27/2014 DEC
 A/Stockholm/23/2015 FEB
 A/Sweden/6/2015 JAN
 A/Florida/63/2014 NOV
 A/New Hampshire/01/2015 JAN
 A/New Hampshire/02/2015 JAN
 A/Trollhattan/4/2015 FEB
 A/Nepal/879/2015 MAR
 A/Nepal/1474/2015 APR
 A/Wisconsin/25/2015 MAR
 A/India/1399/2015 FEB
 A/Stockholm/31/2015 APR
 A/Nepal/574/2015 MAR
 A/India/3498/2015 MAR
 A/India/3389/2015 FEB
 A/India/1368/2015 FEB
 A/India/1268/2015 JAN
 A/India/1247/2015 JAN
 A/India/P1510350/2015 JAN
 A/India/P153017/2015 FEB
 A/India/P157674/2015 APR
 A/India/P154479/2015 MAR
 A/India/P152122/2015 FEB
 A/India/P1510348/2015 MAR
 A/India/3388/2015 FEB
 A/India/P153321/2015 FEB
 A/Indiana/08/2015 JAN
 A/Nepal/560/2015 MAR
 A/Montana/05/2015 FEB
 A/India/3793/2015 MAR
 A/India/1245/2015 JAN
 A/Fukuoka/SDC1/2015 MAR
 A/India/P1510349/2015 FEB
 A/India/P158900/2015 MAR
 A/India/1192/2015 JAN
 A/Myanmer/14M194/2014 JUL
 A/Myanmar/14M272/2014 JUL
 A/Myanmar/14M445/2014 SEP
 A/Myanmar/14M379/2014 AUG
 A/Myanmar/14M233/2014 AUG
 A/Myanmar/14M139/2014 AUG
 A/Myanmar/14M072/2014 JUL
 A/Laos/951/2014 SEP
 A/Jeonbuk/2005/2014 DEC
 A/Washington/59/2014 NOV
 A/Virginia/40/2014 DEC
 A/Laos/952/2014 SEP
 A/Uppsala/5/2015 MAR
 A/Okinawa/14T001/2014 SEP
 A/Nepal/1015/2015 MAR
 A/India/1214/2015 JAN
 A/Ethiopia/66/2014 NOV
 A/Kanagawa/163/2014 SEP
 A/Nonthaburi/375/2014 DEC
 A/Ontario/RV3535/2014 NOV
 A/India/P1510025/2015 JAN
 A/Alaska/37/2014 SEP
 A/Alaska/38/2014 SEP
 A/Nepal/781/2015 MAR
 A/New York/41/2014 DEC
 A/Nepal/1598/2015 APR
 A/Bangladesh/3026/2015 JAN
 A/Mexico/2493/2014 OCT
 A/California/07/2009 APR
0.001
j )
I83M
S29N
M83I
L120F
N29S,T48A
85
S29N, R34Q
Suppl fig. S2j Nakamura et al.,
