# Supplementary material for: Characterization of influenza A(H1N1)pdm09 viruses isolated from Nepalese and Indian outbreak patients in early 2015
Source: Influenza Other Respir Viruses. 2017 Aug 9;11(5):399–403. doi: 10.1111/irv.12469 (PMC5596518; doi:10.1111/irv.12469)
Supplement: Supplementary file 3 [file IRV-11-399-s003.pptx]

## Slide 1
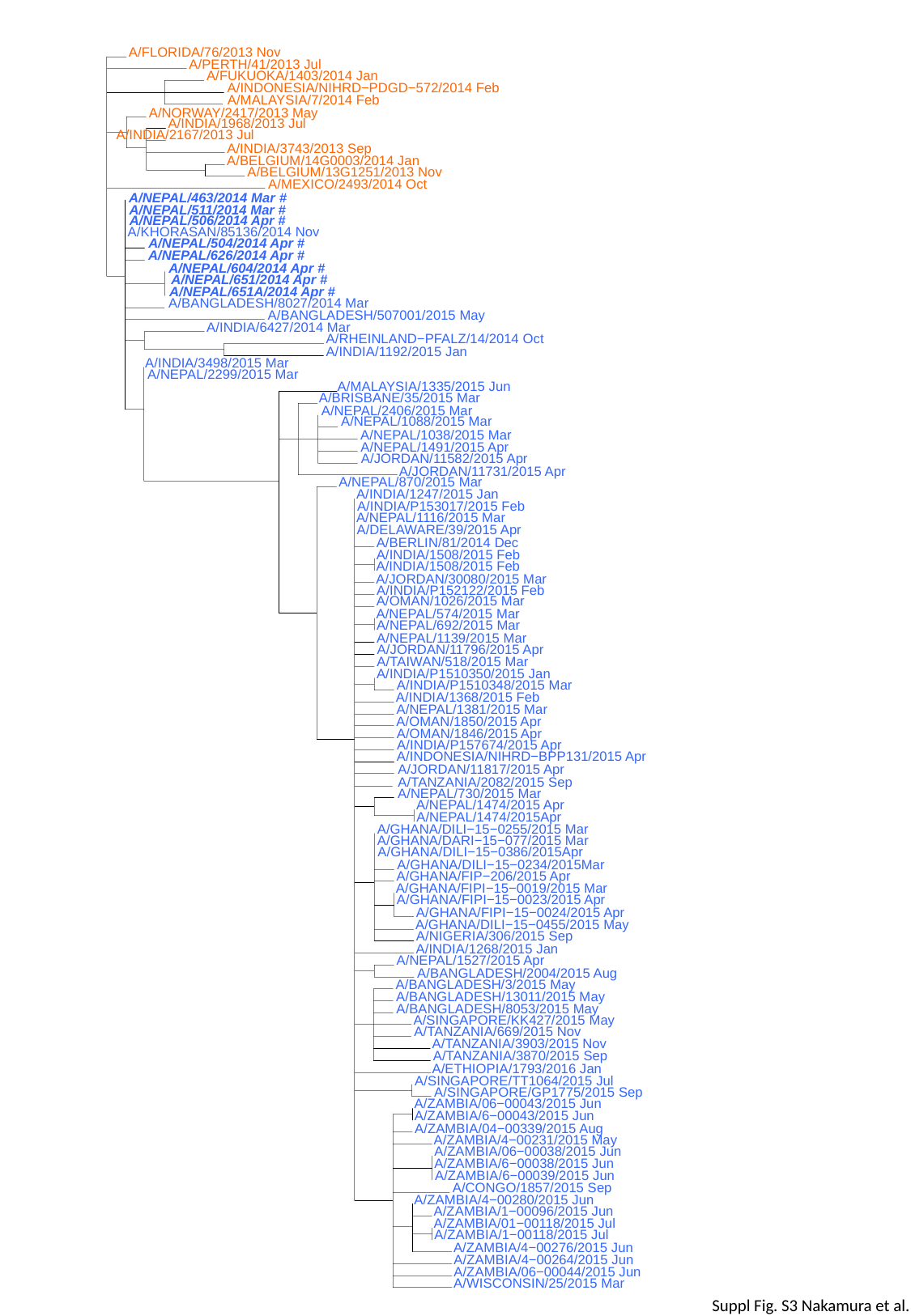

A/FLORIDA/76/2013 Nov
A/PERTH/41/2013 Jul
A/FUKUOKA/1403/2014 Jan
A/INDONESIA/NIHRD−PDGD−572/2014 Feb
	A/MALAYSIA/7/2014 Feb
A/NORWAY/2417/2013 May
A/INDIA/1968/2013 Jul
A/INDIA/2167/2013 Jul
A/INDIA/3743/2013 Sep
A/BELGIUM/14G0003/2014 Jan
A/BELGIUM/13G1251/2013 Nov
A/MEXICO/2493/2014 Oct
A/NEPAL/463/2014 Mar #
A/NEPAL/511/2014 Mar #
A/NEPAL/506/2014 Apr #
A/KHORASAN/85136/2014 Nov
A/NEPAL/504/2014 Apr #
A/NEPAL/626/2014 Apr #
A/NEPAL/604/2014 Apr #
A/NEPAL/651/2014 Apr #
A/NEPAL/651A/2014 Apr #
A/BANGLADESH/8027/2014 Mar
A/BANGLADESH/507001/2015 May
A/INDIA/6427/2014 Mar
A/RHEINLAND−PFALZ/14/2014 Oct
A/INDIA/1192/2015 Jan
A/INDIA/3498/2015 Mar
A/NEPAL/2299/2015 Mar
A/MALAYSIA/1335/2015 Jun
A/BRISBANE/35/2015 Mar
A/NEPAL/2406/2015 Mar
A/NEPAL/1088/2015 Mar
A/NEPAL/1038/2015 Mar
A/NEPAL/1491/2015 Apr
A/JORDAN/11582/2015 Apr
A/JORDAN/11731/2015 Apr
A/NEPAL/870/2015 Mar
A/INDIA/1247/2015 Jan
A/INDIA/P153017/2015 Feb
A/NEPAL/1116/2015 Mar
A/DELAWARE/39/2015 Apr
A/BERLIN/81/2014 Dec
A/INDIA/1508/2015 Feb
A/INDIA/1508/2015 Feb
A/JORDAN/30080/2015 Mar
A/INDIA/P152122/2015 Feb
A/OMAN/1026/2015 Mar
A/NEPAL/574/2015 Mar
A/NEPAL/692/2015 Mar
A/NEPAL/1139/2015 Mar
A/JORDAN/11796/2015 Apr
A/TAIWAN/518/2015 Mar
A/INDIA/P1510350/2015 Jan
A/INDIA/P1510348/2015 Mar
A/INDIA/1368/2015 Feb
A/NEPAL/1381/2015 Mar
A/OMAN/1850/2015 Apr
A/OMAN/1846/2015 Apr
A/INDIA/P157674/2015 Apr
	A/INDONESIA/NIHRD−BPP131/2015 Apr
A/JORDAN/11817/2015 Apr
A/TANZANIA/2082/2015 Sep
A/NEPAL/730/2015 Mar
A/NEPAL/1474/2015 Apr
A/NEPAL/1474/2015Apr
A/GHANA/DILI−15−0255/2015 Mar
A/GHANA/DARI−15−077/2015 Mar
A/GHANA/DILI−15−0386/2015Apr
A/GHANA/DILI−15−0234/2015Mar
A/GHANA/FIP−206/2015 Apr
A/GHANA/FIPI−15−0019/2015 Mar
A/GHANA/FIPI−15−0023/2015 Apr
A/GHANA/FIPI−15−0024/2015 Apr
A/GHANA/DILI−15−0455/2015 May
A/NIGERIA/306/2015 Sep
A/INDIA/1268/2015 Jan
A/NEPAL/1527/2015 Apr
A/BANGLADESH/2004/2015 Aug
A/BANGLADESH/3/2015 May
A/BANGLADESH/13011/2015 May
A/BANGLADESH/8053/2015 May
A/SINGAPORE/KK427/2015 May
A/TANZANIA/669/2015 Nov
A/TANZANIA/3903/2015 Nov
A/TANZANIA/3870/2015 Sep
A/ETHIOPIA/1793/2016 Jan
A/SINGAPORE/TT1064/2015 Jul
A/SINGAPORE/GP1775/2015 Sep
A/ZAMBIA/06−00043/2015 Jun
A/ZAMBIA/6−00043/2015 Jun
A/ZAMBIA/04−00339/2015 Aug
A/ZAMBIA/4−00231/2015 May
A/ZAMBIA/06−00038/2015 Jun
A/ZAMBIA/6−00038/2015 Jun
A/ZAMBIA/6−00039/2015 Jun
A/CONGO/1857/2015 Sep
A/ZAMBIA/4−00280/2015 Jun
A/ZAMBIA/1−00096/2015 Jun
A/ZAMBIA/01−00118/2015 Jul
A/ZAMBIA/1−00118/2015 Jul
A/ZAMBIA/4−00276/2015 Jun
A/ZAMBIA/4−00264/2015 Jun
A/ZAMBIA/06−00044/2015 Jun
A/WISCONSIN/25/2015 Mar
Suppl Fig. S3 Nakamura et al.
