# Supplementary material for: Characterization of influenza A(H1N1)pdm09 viruses isolated from Nepalese and Indian outbreak patients in early 2015
Source: Influenza Other Respir Viruses. 2017 Aug 9;11(5):399–403. doi: 10.1111/irv.12469 (PMC5596518; doi:10.1111/irv.12469)
Supplement: Supplementary file 4 [file IRV-11-399-s004.docx]

Supplementary table S1.

Clinical outcomes of Nepalese patients from whom H1N1pdm was isolated.

| Strain designation | Age | Sex | Clinical severity | Fever | Sore Throat | Coryza | Cough | Breathing difficulty | Chills | Sample collection date |
| --- | --- | --- | --- | --- | --- | --- | --- | --- | --- | --- |
| A/Nepal/0236/2015 | 6y | M | ILI^a)^ | YES | YES | YES | YES | NO | YES | Mar 1, 2015 |
| A/Nepal/0489/2015 | 20y | F | ILI | YES | YES | YES | YES | YES | YES | Mar 6, 2015 |
| A/Nepal/0504/2015 | 31y | M | ILI | YES | YES | YES | YES | NO | NO | Mar 6, 2015 |
| A/Nepal/0560/2015 | 35y | M | SARI^b)^ | YES | NO | NO | YES | YES | NO | Mar 9, 2015 |
| A/Nepal/0574/2015 | 1.5y | F | ILI | YES | YES | YES | YES | NO | NO | Mar 10, 2015 |
| A/Nepal/0664/2015 | 19y | M | SARI | YES | YES | NO | NO | NO | NO | Mar 9, 2015 |
| A/Nepal/0670/2015 | 2y | F | SARI | YES | NO | NO | YES | YES | YES | Feb 27, 2015 |
| A/Nepal/0688/2015 | 11y | F | ILI | YES | NO | NO | NO | NO | YES | Mar 11, 2015 |
| A/Nepal/0692/2015 | 6y | F | ILI | YES | YES | NO | YES | NO | NO | Mar 11, 2015 |
| A/Nepal/0730/2015 | 10y | M | ILI | YES | YES | YES | YES | YES | YES | Mar 12, 2015 |
| A/Nepal/0751/2015 | 28y | F | SARI | YES | YES | YES | YES | YES | NO | Mar 12, 2015 |
| A/Nepal/0781/2015 | 1y | M | ILI | YES | NO | NO | YES | NO | NO | Mar 13, 2015 |
| A/Nepal/0830/2015 | 30y | F | ILI | YES | YES | NO | YES | NO | NO | Mar 15, 2015 |
| A/Nepal/0849/2015 | 5y | M | ILI | YES | NO | YES | YES | YES | YES | Mar 16, 2015 |
| A/Nepal/0870/2015 | 3y | F | ILI | YES | NO | YES | YES | NO | YES | Mar 16, 2015 |
| A/Nepal/0871/2015 | 43y | F | SARI | YES | NO | NO | YES | YES | YES | Mar 16, 2015 |
| A/Nepal/0873/2015 | 55y | F | ILI | YES | YES | NO | YES | NO | NO | Mar 16, 2015 |
| A/Nepal/0879/2015 | 9m | M | ILI | YES | NO | NO | YES | NO | NO | Mar 16, 2015 |
| A/Nepal/0880/2015 | 9m | M | ILI | YES | NO | NO | YES | NO | NO | Mar 16, 2015 |
| A/Nepal/0931/2015 | 3y | M | ILI | YES | YES | YES | YES | NO | NO | Mar 17, 2015 |
| A/Nepal/0989/2015 | 18y | M | ILI | YES | YES | YES | YES | YES | YES | Mar 18, 2015 |
| A/Nepal/1015/2015 | 73y | M | ILI | YES | NO | YES | YES | NO | YES | Mar 18, 2015 |
| A/Nepal/1022/2015 | 11y | M | ILI | YES | NO | YES | YES | YES | YES | Mar 19, 2015 |
| A/Nepal/1038/2015 | 30y | F | SARI | YES | YES | YES | YES | YES | YES | Mar 19, 2015 |
| A/Nepal/1088/2015 | 20y | M | ILI | YES | NO | NO | YES | NO | NO | Mar 13, 2015 |
| A/Nepal/1116/2015 | 5y | M | ILI | YES | YES | NO | YES | NO | YES | Mar 22, 2015 |
| A/Nepal/1120/2015 | 24y | M | ILI | YES | YES | YES | YES | NO | YES | Mar 22, 2015 |
| A/Nepal/1139/2015 | 9y | M | ILI | YES | YES | YES | YES | NO | YES | Mar 22, 2015 |
| A/Nepal/1253/2015 | 52y | M | ILI | YES | YES | YES | YES | YES | NO | Mar 26, 2015 |
| A/Nepal/1326/2015 | 19y | F | ILI | YES | YES | NO | YES | NO | NO | Mar 24, 2015 |
| A/Nepal/1361/2015 | 42y | M | ILI | YES | YES | YES | YES | NO | NO | Mar 30, 2015 |
| A/Nepal/1381/2015 | 30y | M | ILI | YES | YES | YES | YES | YES | YES | Mar 31, 2015 |
| A/Nepal/1395/2015 | 3y | M | ILI | YES | YES | YES | YES | NO | NO | Apr 1, 2015 |
| A/Nepal/1400/2015 | 2.5y | F | ILI | YES | NO | YES | YES | NO | NO | Apr 1, 2015 |
| A/Nepal/1474/2015 | 23y | M | ILI | YES | YES | NO | YES | NO | NO | Apr 6, 2015 |
| A/Nepal/1475/2015 | 25y | F | ILI | YES | YES | YES | YES | YES | YES | Apr 6, 2015 |
| A/Nepal/1478/2015 | 9y | M | ILI | YES | YES | YES | YES | NO | NO | Apr 6, 2015 |
| A/Nepal/1491/2015 | 37y | F | SARI | NO | NO | YES | YES | YES | YES | Apr 7, 2015 |
| A/Nepal/1527/2015 | 61y | F | ILI | YES | NO | YES | YES | YES | YES | Apr 10, 2015 |
| A/Nepal/1544/2015 | 58y | M | SARI | YES | YES | YES | YES | YES | NO | Apr 12, 2015 |
| A/Nepal/1586/2015 | 32y | F | ILI | YES | YES | YES | YES | YES | YES | Apr 15, 2015 |
| A/Nepal/1598/2015 | 5y | M | ILI | YES | YES | NO | YES | NO | NO | Apr 14, 2015 |
| A/Nepal/1600/2015 | N.A. | F | ILI | YES | YES | NO | YES | NO | YES | Apr 14, 2015 |
| A/Nepal/1619/2015 | 2.5y | M | ILI | YES | YES | YES | YES | YES | YES | Apr 17, 2015 |
| A/Nepal/1630/2015 | 23y | M | ILI | YES | YES | YES | NO | YES | YES | Apr 15, 2015 |
| A/Nepal/1636/2015 | 13y | M | SARI | YES | NO | YES | YES | YES | NO | Apr 19, 2015 |
| A/Nepal/1647/2015 | 78y | F | SARI | YES | YES | YES | YES | YES | NO | Apr 19, 2015 |
| A/Nepal/1738/2015 | N.A. | F | SARI | YES | YES | YES | YES | YES | YES | Apr 19, 2015 |
| A/Nepal/1739/2015 | 80y | F | SARI | YES | YES | YES | YES | YES | YES | Apr 19, 2015 |
| A/Nepal/1746/2015 | 43y | M | SARI | YES | YES | YES | YES | YES | YES | Apr 19, 2015 |

^a)^ ILI: Influenza like illness presenting as fever, sore throat, and cough.

^b)^ SARI: Severe acute respiratory infection; breathing difficulty was observed in addition to ILI symptoms.
