# Supplementary material for: Characterization of influenza A(H1N1)pdm09 viruses isolated from Nepalese and Indian outbreak patients in early 2015
Source: Influenza Other Respir Viruses. 2017 Aug 9;11(5):399–403. doi: 10.1111/irv.12469 (PMC5596518; doi:10.1111/irv.12469)
Supplement: Supplementary file 5 [file IRV-11-399-s005.docx]

Supplementary table S2. Genetic features of the internal genes of outbreak isolates

| Isolate |  |  |  | Amino acid substitutions^a)^ in internal genes | | | | | | | | |
| --- | --- | --- | --- | --- | --- | --- | --- | --- | --- | --- | --- | --- |
|  | Sample date | Clinical outcome |  | M1 | M2 | NP | PA | PB1 | PB1-F2 | PB2 | NS1 | NS2 |
| A/Nepal/560/2015 | 2015/03/09 | SARI^c)^ |  | Q208K |  | A22T  M105T | E227K  A337T  R361K |  |  | D65G  R299K  R368K  S453T |  | M83I |
| A/Nepal/574/2015 | 2015/03/10 | ILI^b)^ |  | Q208K |  | A22T  M105T | R361K |  |  | R299K  S453T | D53N | M83I |
| A/Nepal/781/2015 | 2015/03/13 | ILI |  |  |  | A22T  S314C  T472A | S522G |  | Q5P | R299K |  |  |
| A/Nepal/879/2015 | 2015/03/16 | ILI |  | Q208K |  | A22T  M105T | S186N  R361K | G216S |  | D146G  R299K  S453T |  | Q68X  W78X  L79X  M83X  T98X |
| A/Nepal/1015/2015 | 2015/03/18 | ILI |  |  |  | A22T  M105T  S486X^d)^ |  | N213S |  | T76I  S453P | S42X |  |
| A/Nepal/1474/2015 | 2015/04/06 | ILI |  | Q208K |  | A22T  M105T | I30X  R361K |  |  | R299K  S453T  M756X |  | R77X  W78X  L79X  M83I |
| A/Nepal/1598/2015 | 2015/04/14 | ILI |  |  | T11I | A22T  R31X |  |  |  | N195D |  |  |
| A/India/P152122/2015 | 2015/02/13 | SARI^c)^, death |  | Q208K |  | A22T  M105T | I61V  R361K |  |  | Q288L  R299K  S453T | M93I |  |
| A/India/P153017/2015 | 2015/02/23 | SARI, death |  | Q208K |  | A22T  M105T | R361K | I364V |  | R299K  S453T | D207N |  |
| A/India/P153321/2015 | 2015/02/25 | SARI, death |  | Q208K |  | A22T  M105T | P331S  R361K |  |  | M64I  R299K  S453T | M98I  E125G | L120F |
| A/India/P154479/2015 | 2015/03/13 | SARI, recovery |  | Q208K |  | A22T  M105T | R361K | N212D |  | R299K  S453T  S590N |  |  |
| A/India/P157674/2015 | 2015/04/02 | SARI, recovery |  | Q208K |  | A22T  M105T  S450N | R361K | I322V |  | R299K  S453T |  |  |
| A/India/P158900/2015 | 2015/03/30 | SARI, recovery |  | Q208K |  | A22T  M105T | R361K |  |  | T106A  E249K  R299K  R368K  S453T | M106X |  |
| A/India/P1510025/2015 | 2015/01/09 | SARI, recovery |  |  |  | A22T | V330I  K361R  S522G | D618B |  |  |  |  |
| A/India/P1510348/2015 | 2015/03/04 | SARI, recovery |  | Q208K |  | A22T  M105T | R212C  R361K |  |  | L163M  R299K  S453T |  |  |
| A/India/P1510349/2015 | 2015/02/03 | SARI, recovery |  | Q208K |  | A22T  M105T | A337T  R361K |  |  | K61R  R299K  R368K  S453T |  |  |
| A/India/P1510350/2015 | 2015/01/31 | SARI, recovery |  | Q208K |  | A22T  M105T |  |  |  | R299K  S453T  M570I  D680B |  | N92S |

^a)^ As compared to the consensus sequence generated from over 600 sequences deposited to GISAID database from worldwide in 2014/15 season.

^b)^ ILI: Influenza like illness presenting as fever, sore throat, and cough.

^c)^ SARI: Severe acute respiratory infection; breathing difficulty was observed in addition to ILI symptoms.

^d)^ ‘X’ indicates a mixed population of amino acids at the designated position.
